# Chaos analysis of the brain topology in first-episode psychosis and clinical high risk patients

**DOI:** 10.3389/fpsyt.2022.965128

**Published:** 2022-10-13

**Authors:** Alexandra I. Korda, Christina Andreou, Mihai Avram, Heinz Handels, Thomas Martinetz, Stefan Borgwardt

**Affiliations:** ^1^Translational Psychiatry, Department of Psychiatry and Psycotherapy, University of Lübeck, Lübeck, Germany; ^2^Institute of Medical Informatics, University of Lübeck, Lübeck, Germany; ^3^Institute for Neuro- and Bioinformatics, University of Lübeck, Lübeck, Germany

**Keywords:** first-episode psychosis, clinical high risk, brain sMRI image, biomarkers, chaos analysis, Lyapunov exponent, wavelet transformation, gray matter topology

## Abstract

Structural MRI studies in first-episode psychosis (FEP) and in clinical high risk (CHR) patients have consistently shown volumetric abnormalities in frontal, temporal, and cingulate cortex areas. The aim of the present study was to employ chaos analysis for the identification of brain topology differences in people with psychosis. Structural MRI were acquired from 77 FEP, 73 CHR and 44 healthy controls (HC). Chaos analysis of the gray matter distribution was performed: First, the distances of each voxel from the center of mass in the gray matter image was calculated. Next, the distances multiplied by the voxel intensity were represented as a spatial-series, which then was analyzed by extracting the Largest-Lyapunov-Exponent (lambda). The lambda brain map depicts thus how the gray matter topology changes. Between-group differences were identified by (a) comparing the lambda brain maps, which resulted in statistically significant differences in FEP and CHR compared to HC; and (b) matching the lambda series with the Morlet wavelet, which resulted in statistically significant differences in the scalograms of FEP against CHR and HC. The proposed framework using spatial-series extraction enhances the between-group differences of FEP, CHR and HC subjects, verifies diagnosis-relevant features and may potentially contribute to the identification of structural biomarkers for psychosis.

## Introduction

Patterns of pathological alterations of the brain associated with illness emergence and progression have been a major interest of research in psychotic disorders (1). Abnormalities in brain gray matter (GM) topology have been consistently observed in schizophrenia; these appear to be present already at the first-episode of psychosis (FEP) and even in subjects at clinical high risk (CHR) patients (2–8). Abnormalities in cortical surface areas and cortical thickness have been consistently observed in schizophrenia as well, including in FEP (9, 10). Drobinin et al. (11) reported that in youth at risk for mental illness displayed an overall trend toward lower cortical folding across all brain regions. Results from previous studies of cortical folding in schizophrenia showed abnormal folding in large regions of the cerebral cortex across several independent samples of patients with schizophrenia, mostly affecting frontotemporal regions (12–14) and increased structural variability of regional measures of brain morphology (15, 16). These conceivably relate to abnormal topological organization of structural brain networks in schizophrenia, which has been reported in several studies (17–19), and may affect mental skills and sensorimotor functions (20); for example, neuroanatomical alterations in the medial prefrontal cortex and hippocampus were associated with abnormalities of the recognition of negative emotion at baseline in CHR patients (21).

As the GM morphology is inherently complex, chaos and nonlinear dynamics analyses of these spatial data are suitable mathematical techniques for extracting their informative statistical properties. Chaos and nonlinear dynamics have been increasingly reported as effective computational methods for analyzing complex data in medicine and biology (22). Wahman et al. (23), suggested that psychiatric disorders are better accounted for by the nonlinear dynamics of chaos theory than by a unidirectional vector model of cause to effect. Several mental disorders are thought to follow instability patterns captured from chaos theory, resulting in complicated emotional/cognitive and interpersonal interactions and phenomenological presentations (24, 25). In patients with schizophrenia, chaos theory has been applied at the behavioral level to quantify higher interdependencies in the response sequences generated by patients in a choice task compared to healthy controls (26).

The aim of the present study was to employ chaos analysis for the identification of brain topology changes in psychosis based on structural MRI. We hypothesized that the nonlinear dynamics of brain GM topology in FEP and CHR is different from that of HC. We introduce the term of GM topology for analysis of GM changes combining two features, voxel distance from the center of mass and voxel intensity. We aimed to compare GM topology including cortical surface between FEP, CHR and HC subjects. Our ultimate goal was to analyze the chaotic dynamic of the GM topology and its usefulness for marking progression of the illness. To this purpose, we investigated GM topology patterns on the group level. We compared the complexity of the GM topology across 77 FEP, 73 CHR and 44 HC by transforming structural magnetic resonance imaging (sMRI) GM maps into spatial-series. The term spatial-series refers to the distribution of the weighted distance by voxel intensity from the GM center of the mass; this approach, i.e., the conversion of images into sequences for application of time-series analysis has been utilized for solving several problems in image data mining (27), including investigation of abnormal brain folding in Alzheimer’s disease and aging (28). In addition, a previous study reported differences on the complexity of brain folding in aging by transforming the sMRI scans into spatial series and comparing the Largest-Lyapunov-Exponent values between patients and controls (22).

We investigated the lambda to determine the chaos and nonlinear dynamics of spatial-series data of GM topology in psychotic disorders extracted from structural brain MRI. Lambda expresses the divergence of the small distances over voxel location, and thus can be used as a quantitative measure of geometry and curvature and therefore topological complexity of assessed brain regions. In order to quantify and compare the complexity of GM distribution between FEP, CHR, and HC, we used continuous wavelet transformation (CWT) to decompose the lambda series into their frequency (“multi-scale”) components representing the structure relief. In the results presented below, the term “scale” refers to the inverse frequency; scales are represented by a scalogram, in which the *x*-axis corresponds to spatial points (i.e., assessed voxels, represented as a spatial series) and the y-axis to their scale as defined above. We show that this approach provides interesting insights for differentiation of FEP and CHR from HC, and FEP from CHR at the group level; beyond those provided by standard volumetric comparisons by means of voxel-based morphometry (VBM).

## Materials and methods

### Study participants

In this study, sMRI scans of 194 subjects were used, 77 FEP patients, 73 CHR subjects and 44 healthy controls (HC). Subjects were scanned using a SIEMENS MAGNETOM VISION 1.5T scanner (Erlangen, Germany) at the University Hospital Basel, Switzerland. The current analyses are based on data from patients included in the early detection of psychosis project (FePsy) at the Department of Psychiatry of the University of Basel (29) between November 2008 and April 2014. The Basel Screening Instrument for Psychosis (BSIP) was used for assessment of CHR and FEP status. The BSIP is a 46-item instrument based on variables that have been shown to be risk factors for early symptoms of psychosis such as DSM-III-R – “prodromal symptoms,” social decline, drug abuse, previous psychiatric disorders, or genetic liability for psychosis (30). CHR status was defined based on the presence of either attenuated psychotic symptoms, brief limited intermittent psychotic symptoms, or having a first- or second-degree relative with a psychotic disorder plus at least 2 additional risk factors for psychosis. FEP status was defined according to criteria for transition to psychosis by Yung et al. (31). The study was approved by the local ethics committee of the University of Basel and written informed consent is obtained from each participant. The study was conducted in accordance with the Declaration of Helsinki.

A three-dimensional volumetric spoiled gradient recalled echo sequence generated 176 contiguous, 1-mm-thick sagittal slices. Imaging parameters were time-to-echo, 4ms; time-to-repetition, 9.7ms; flip angle, 12°; matrix size, 200 × 256; field of view, 25.6 cm × 25.6 cm matrix; voxel dimensions, 1.28 mm × 1 mm × 1 mm. Inclusion and exclusion criteria were described in Borgwardt et al. (32).

### Magnetic resonance imaging data processing

After inspection for artifacts and gross abnormalities, the T1-weighted images were segmented into gray matter (GM), white matter (WM), and cerebrospinal fluid (CSF) tissue maps in native space with the CAT12 toolbox^1^, an extension of the SPM12 software package (Wellcome Department of Cognitive Neurology, London, England). In detail, the CAT12 toolbox extends the unified segmentation model consisting of MRI field intensity inhomogeneity correction, spatial normalization and tissue segmentation in several preprocessing steps to further improve the quality of data preprocessing. Initially, the Optimized Blockwise Nonlocal-Means filter proposed by Coupe at al. (33) was applied to the MRI scans using the Rician noise adaption introduced in Wiest-Daesslé et al. (34) to increase the signal-to-noise ratio in the data. The usual strip artifacts in modulated images are greatly reduced by the default internal interpolation setting “Fixed 1 mm” in CAT12 toolbox. Subsequently, an adaptive maximum a posteriori segmentation approach (35) extended by partial volume estimation (36) was employed to separate the MRI scans into GM, WM, CSF tissue. The segmentation step was finished by applying a spatial constraint to the segmented tissue probability maps based on a hidden Markov Random Field model (37) that removed isolated voxels which were unlikely to be a member of a certain tissue class and closed gaps in clusters of connected voxels of a certain class, resulting in a higher signal-to-noise ratio of the final tissue probability maps. The strength of the filters is automatically determined by estimating the residual noise in the image. The original voxels are projected into their new location in the warped images preserving the volume of a particular tissue within a voxel (i.e., produced by affine transformation (global scaling) and non-linear warping (local volume change)). All scans were reviewed by a neuroradiologist to rule out clinically relevant abnormalities, data did not present any artifacts. Each participant’s GM, WM, and CSF maps were registered to stereotactic standard space. Next, for each subject, the modulated, warped, GM image (mwp1*) with an isotropic voxel size of 1.5 mm, was selected for the extraction of the spatial-series.

**FIGURE 1:**
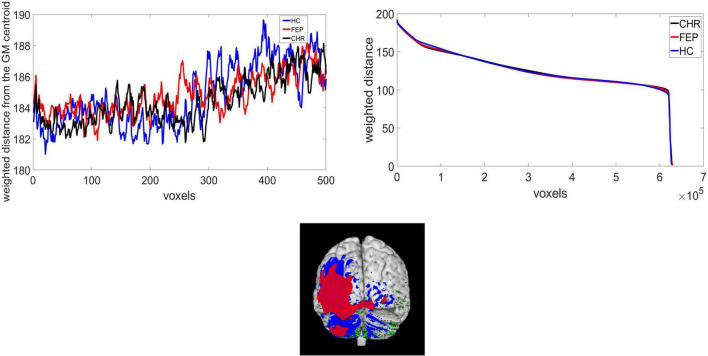
**(A)** Weighted distance from the GM center of the mass and voxels, extracted from HC, CHR, and FEP group, **(B)** the sorted weighted distance and **(C)** the selected voxels for CHR(red), FEP(blue), and HC(green).

The function *centerofMass* in MATLAB 2020b was used to get the center of the mass of the GM. This function finds the gray-level-weighted center of mass of a N-dimensional numerical array (3-dimensional array of size 121 × 145 × 121 in this case). In the Supplementary Table 1 the mean and standard deviation of the center of the mass of each mwp1* brain image across groups for the x, y, z coordinates are presented. *T*-test with 5% significance level was performed for the group comparison. Statistically significant differences were identified for the x coordinate between FEP and CHR groups. To capture geometric changes in brain regions we calculated the weighted distance of the GM center of the mass with the voxel intensities (distance in mm × voxel intensity). The histogram of the intensities for the warped and modulated images from all subjects is presented in Supplementary Figure 1. Top-weighted distances correspond to voxels that either belong to the cortical surface or slightly close to the center of the mass of the GM. For example, one point with intensity 1 and distance from the GM center of the mass 3 has the same weighted distance as one point with intensity 3 and distance 1. Though, the intensities are not restricted to [0,1] and the distance in mm outweighs intensity. In Supplementary Figure 2 we present the distance values in (a) and weighted distance values in (b) for one HC subject. It is observed that the highest distances are located in the temporal and occipital lobe (red color in a). However, high weighted distances are also located at the middle frontal gyrus, precuneus, left parietal and left caudate. We added some examples of the differences between distance and weighted distance in frontal lobe and cerebellum lobe in Supplementary Figures 2C,D) which explains how the weighted distance is affected by the intensities and the distances. In Supplementary Figure 2C) the upper graph shows the distance at the middle frontal gyrus (117.119) and the lower graph shows the weighted distance (160.431). In Supplementary Figure 2D) the left cerebellum exterior presents distance equal to 193.315 (upper graph) and weighted distance equal to 122.072 (lower graph). While the cerebellum has high distance the weighted distance of the middle frontal gyrus is higher. Reducing the computational costs, the weighted distances were sorted from the highest to lowest value. After experimentation, we concluded that the highest 5,000 voxels, expressing almost 1% of the non-zero voxels in the GM image, may be used for the identification of brain topology differences of FEP and CHR patients. We ran the analysis using 1,000 to 5,000 voxels with a step of 1,000 voxels; significant results were identified selecting 5,000 voxels. We were interested in a method able to function under computational restrictions, which is why we chose only 1% of the voxels (5,000) for analysis. In Figure 1A), a presentation of the mean weighted distance across every group for 500 voxels is shown. The measure was calculated with the same initial condition across subjects, starting from the same voxel and ends at the same voxels. The sorted mean weighted distance from each group is presented in Figures 1B,C). FEP and CHR overlay most of the brain regions (i.e., left precentral, frontal, rolandic, insula, olfactory, amygdala, and heschl left, cingulum, hippocampus, temporal and occipital) which indicates that the selected voxels based on the higher weighted distances are spatial dependent. Next, we analyzed the complexity of the brain topology to identify the stage of psychosis using the lambda and the wavelet transformation.

### Largest-Lyapunov-Exponent

One of the most well-known method for quantitative measures of chaos is the Largest-Lyapunov-Exponent (lambda). A positive lambda expresses sensitive dependence on initial conditions for a dynamical system. A positive lambda presents the average rate over the whole attractor, at which two nearby trajectories become exponentially separate with time evolution (38). A practical numerical technique for calculating lambda is the method developed by Rosenstein et al. (39), which works well with small datasets and is robust to changes in the embedding dimension, reconstruction delay, and noise level (40). In brief, let *x_i_* denote the spatial-series of the path free sorted distances extracted from the brain sMRI. If it is assumed that the given spatial-series provides an observation of a dynamical system where the parameter time is replaced by the voxel intensity and distance from the center of the mass combined, then according to the theorem of Takens (40), the trajectory of the attractor of the system whose states evolves with spatial location over a state-space and predict the interactions over location between multiple voxel intensities can be described by a matrix *X*. Takens Theorem is not restricted to time series. It is necessary that the series is determined by a trajectory in a finite state-space. Even if this is not exactly given, one can apply the procedure of determining lambda and regard lambda as a feature describing structural aspects of the cortex. Each row, *X_k_*, of the matrix is a state space vector:


(1) $$X_{k}=\left[x_{k},x_{k+\tau },x_{k+2\,\tau },\ldots ,x_{k+\left(m-1\right)\,\tau }\right]$$

where *k* = 0,1,…,*M*−1, *M* = *N*−(*m*−1)τ, N is length of the spatial-series, τ and *m* are the embedding delay and the embedding dimension, respectively (41, 42).

After the state space reconstruction, the lambda can be defined using the following equation:


(2) $$d\,\left(t\right)=d\,\left(0\right)\,e^{\lambda_{1}\,t}$$

where λ_1_ is the lambda value, *d*(*t*) is the average divergence at the voxel *t*, and *d*(0) is a constant that normalizes the initial separation.

Lambda can be estimated using the matrix *X* of the reconstructed state space as in (38). A spatial-dependent value of lambda, λ_1_(*k*), where *k* the target voxel and *T* the distance between voxels in the state space, can be estimated as:


(3) $$\lambda_{1}\,\left(k\right)=\frac{\left\langle l\,n\,d\,\left(k\right)\right\rangle -\left\langle l\,n\,d\,\left(k-1\right)\right\rangle }{T},k=1$$

**FIGURE 2:**
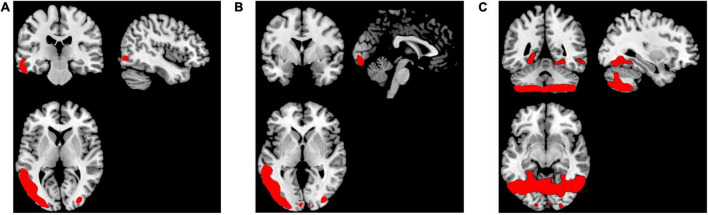
Representation of the smoothed mean lambda map of the top-weighted distances in red for **(A)** FEP, **(B)** CHR, and **(C)** HC.

To perform localization, we first sorting the weighted distance of each voxel and its coordinates were stored in a variable. Then, we selected 5,000 lambda values which mapped back to GM images using the stored coordinates. The lambda of voxels that were not selected as the top-weighted voxels is set to 0. The smoothed mean lambda map using a Gaussian kernel *8 x 8 x 8* mm for FEP patients is presented in Figure 2A), for CHR subjects in Figure 2B) and for HC subjects in Figure 2C). The images were represented on MNI space by using a GM template in the MRIcron toolbox. In the Supplementary Table 2 the mean values of the lambda in each group were calculated for the selected voxels using the aal.nii template in the MRIcron.

We performed a voxel-wise two-sample *t*-test in SPM12 toolbox for multiple comparisons (FWE <0.05, cluster-defining threshold of *p* < 0.001) to compare the lambda value maps between (a) FEP vs. HC, (b) CHR vs. HC and (c) FEP vs. CHR (see Supplementary Tables 3, 4). The demographic variables: Education (years), years of Smoking (Cigarettes per Day), alcohol, age, and sex were used separately as covariates in (a), (b), and (c) comparisons.

### Wavelet transformation

Wavelet transform (WT) employs a fully scalable modulated window, which provides an extensively tested solution to the windowing function selection problem in frequency-related (scale-related) signal processing methodologies (43). The window slides across the signal, and for every position a spectrum is calculated. The procedure is then repeated at a multitude of scales, providing a signal representation with multiple spatial-scale resolutions. It does not only inform us about which scales are present in a signal, but also at which geometrical point these scales occur. This allows for good point resolution for high-scale events, as well as good scale resolution for low-scale events, which is a combination of properties particularly well-suited for real signals. The rationale of the WT approach is that, firstly, the signal is “viewed” at a large scale/window and “large” features are analyzed and then the signal is “viewed” at smaller scales, in order to analyze “smaller” features.

**FIGURE 3:**
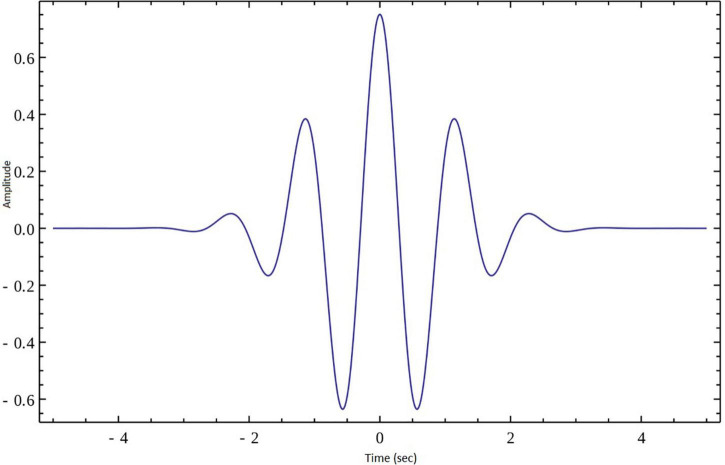
Morlet wavelet.

In the present work continuous wavelet transform (CWT) was used for extracting features from lambda series obtained from the three types of groups that were described above. CWT was used to decompose the lambda series into their frequency components and the statistical features of the CWT coefficients were computed in the spatial domain. A CWT with a complex Morlet as mother function was used, see Figure 3. The WT of a 1-dimensional (1D) series has two dimensions. This 2-dimensional (2D) output of the WT provides the spatial-scale representation of the original series in the form of a “scalogram” plane. The two dimensions of a scalogram are the geometrical points and the scales. Each value (wavelet coefficient) in the scalogram plane represents the correlation of the lambda series with the Morlet wavelet on the respective point and scale pair.

In this study, different scales of the wavelet were examined, ranged from 20 to 200. Scales in the range of 100 to 200 do not provide further information but for the 500 first and last voxels, fact that complicates the representation of the differences between groups (see Supplementary Figure 3). Group-comparisons with corrected p-values were performed using the scalograms from 20 to 200 scales, concluding that 100 scales reveal significant differences and a better representation for each group. T-tests for the selected scales were conducted with the significance level set at *p* < 0.05 (corrected with FDR < 0.05), for the following group comparisons: (i) FEP vs. HC, (ii) FEP vs. CHR, and (iii) CHR vs. HC. A surface overlay of the mean correlation of each group with Morlet wavelet was applied to represent the statistically significant differences in scales. Gyrification index was calculated using the CAT12 toolbox (surface-based morphometry analysis) and analyzed by applying general linear models for group comparisons. We calculate the gyrification index based on absolute mean curvature (44), using a 20mm kernel as suggested in the CAT12 manual, for folding data.

**FIGURE 4:**
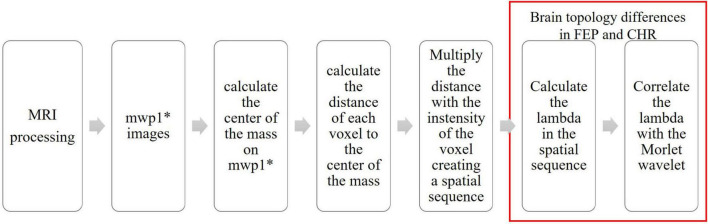
Flowchart of the processing steps.

The full workflow of analyses is presented in Figure 4.

## Results

### Sociodemographic characteristics

There were no significant differences between FEP and HC with respect to age and alcohol consumption. For the comparison between FEP and CHR, there were no significant differences with respect to age, years of education, smoking, alcohol consumption and sex. There were significant differences between FEP and CHR with respect to BPRS total, BPRS Negative and Positives Symptoms and SANS total and between CHR and HC with respect to sex (see **Table 1**).

**TABLE 1:** Group comparison was investigated using 1-way ANOVA for continuous and χ^2^ test for categorical data.

*One-Way ANOVA (Welch’s)*		
	*F*	*P*
**FEP vs HC**		
Age	1.63	0.204
Education (Years)	19.26	< 0.001
Smoking (Cigarettes per Day)	28.18	< 0.001
**CHR vs HC**		
Age	2.082	0.152
Education (Years)	10.654	0.002
Smoking (Cigarettes per Day)	8.634	0.004
**FEP vs. CHR**		
Age	3.118	0.056
Education (Years)	0.165	0.849
Global Assessment of Functioning (GAF)	46.875	< 0.001
BPRS_Positive_Symptoms	19.498	< 0.001
BPRS_Negative_Symptoms	22.494	< 0.001
BPRS_total	462.930	< 0.001
SANS_total	128.957	< 0.001
Smoking (Cigarettes per Day)	2.418	0.102
***χ^2^ Tests***		
**FEP vs. HC**		
Sex	13.6	< 0.001
Alcohol	9.60	0.008
**CHR vs. HC**		
Sex	17.7	< 0.001
Alcohol	0.14	0.710
**FEP vs. CHR**		
Sex	0.431	0.511
Alcohol	2.80	0.246

Note: BPRS, Brief Psychiatric Rating Scale; GAF, Global Assessment of Functioning; SANS, Scale for the Assessment of Negative Symptoms.

### Localization

**FIGURE 5:**
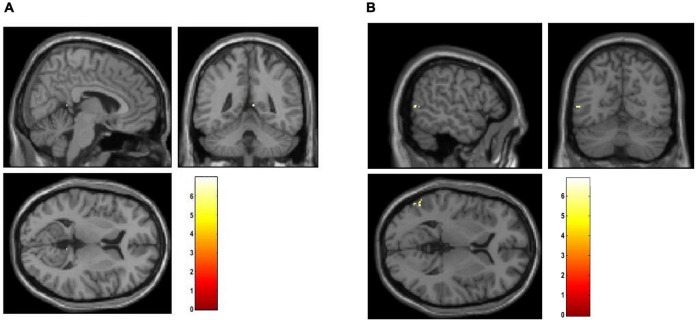
Visualization of between-group comparison results using the mean lambda maps represented. Two sample t-test was applied with age, sex and smoking as covariates in **(A)** FEP > HC, and **(B)** CHR > HC. The corrected *p*-values (FWE < 0.05) are presented.

The smoothed mean lambda map for FEP patients is presented in Figure 2A), for CHR subjects in Figure 2B) and for HC subjects in Figure 2C) for better representation. Common regions are captured in FEP and CHR subjects (e.g., frontal, temporal, and cingulate cortex areas, hippocampus, cerebellum and vermis). In the Supplementary Table 2, the mean values of the lambda in brain regions were calculated using the aal.nii template in the MRIcron. Fact that leads the assumption that sorted weighted distances are spatial dependent across brain regions. In FEP vs. HC comparison, age, sex, and smoking have an effect in temporal lobe (FEP > HC), there is no effect of the years of education and alcohol. Applying all the demographic variables as covariates statistically significant differences were present in the temporal pole, right posterior cingulate gyrus, lingual gyrus and fusiform (FEP > HC, see Supplementary Table 3). Statistically significant differences were present in occipital and temporal lobe for CHR compared to HC (CHR > HC, see Supplementary Table 4). The comparison of CHR with HC is not affected by the demographic variables. BPRS_total, BPRS_positive, BPRS_negative, GAF and SANS scores were used as additional covariates in comparison c). No clusters were identified in between-group comparison results for CHR and FEP. FWE-corrected *p*-values with a threshold of 10 voxels are presented in Figure 5.

For comparison, voxel-based morphometry (VBM) analyses were performed in SPM12 toolbox on smwp1* images to identify volumetric brain differences between groups (a) FEP vs. HC and (b) CHR vs. HC and (c) FEP vs. CHR. Demographic variables, Education (years), years of Smoking (Cigarettes per Day), alcohol, age and sex were used as covariates. BPRS_total, BPRS_positive, BPRS_negative, GAF and SANS scores were used as additional covariates in comparison c). There were significant differences for corrected *p*-values (FWE < 0.05, cluster-defining threshold of *p* < 0.001 and extent threshold voxels of 10) in comparison (a) (details in Supplementary Table 5). There were no significant differences for corrected *p*-values (FWE < 0.05, cluster-defining threshold of *p* < 0.001) in (b) and (c) comparisons.

### Multi-scale analysis

**FIGURE 6:**
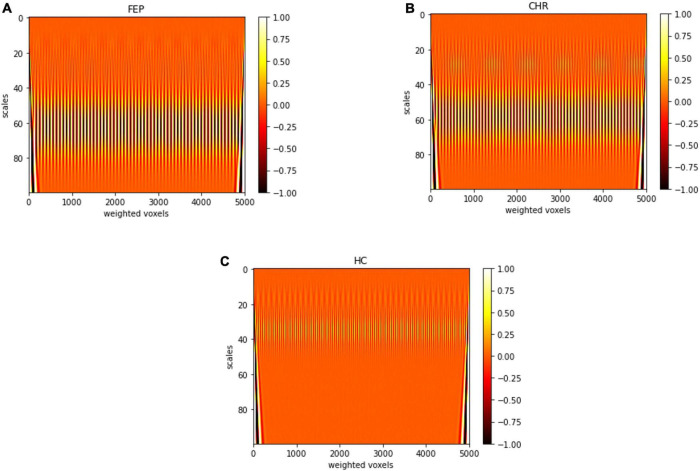
Scalogram of one **(A)** FEP, **(B)** CHR, and **(C)** HC subject for 100 scales. Sampled voxels are represented in *x*-axis and scales in *y*-axis, the colors represent the correlation with the Morlet wavelet deep red color corresponds to lower and white color to higher correlation.

In Figure 6, representative scalograms of one CHR subject, one FEP and one HC are presented for 100 scales. There are positive “matches” (correlation) and negative “matches” with the Morlet wavelet; the color blue corresponds to high negative and the white color to high positive correlation. Such scalograms can be used to better understand the dynamical behavior of a system and can also be used for distinguishing signals produced by different systems in frequency domain. FEP and CHR patients presented a common pattern such as the one shown in Figures 6A,B), i.e., mostly higher absolute correlation with the Morlet wavelet in large scales between 20 and 80. HC subjects presented a common pattern such as the one in Figure 6C), i.e., higher absolute correlation with Morlet wavelet in large scales between 20 and 50. See Supplementary Figure 2 for individual scalograms of all analyzed FEP, CHR and HC. Results of the multi-scale analysis were generated in approximately 1 hour for all the subjects together using parallelization on a high-performance computer (OMICS cluster by the University of Luebeck).

**FIGURE 7:**
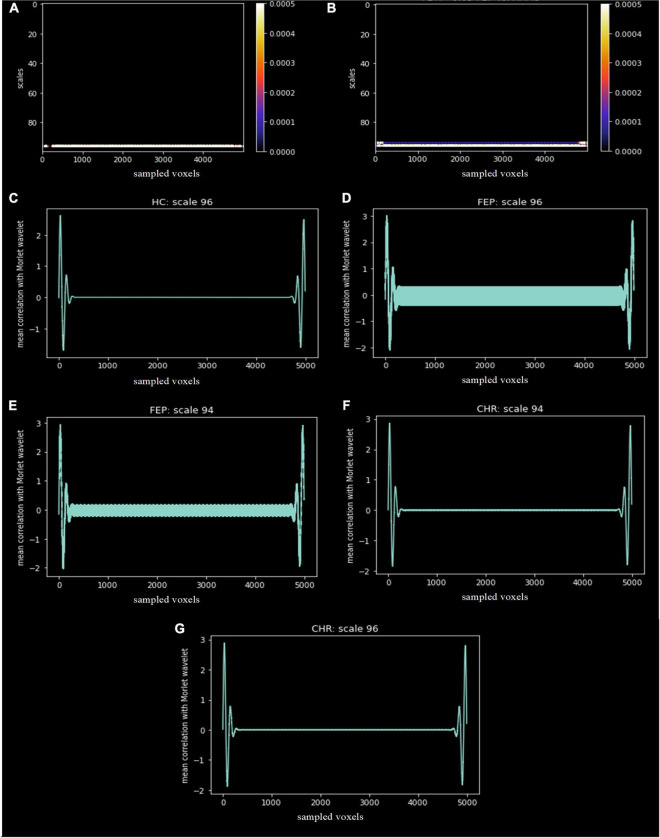
Statistically significant differences, corrected with FDR < 0.05, between groups **(A)** FEP vs. HC and **(B)** FEP vs. CHR. The x-axis represents the sampled voxels, the y-axis represents the scales and the colors represent the value of the corrected p-values. The colorbar shows the range of the corrected *p*-values in this graph. The mean correlation of the Morlet wavelet with the lambda values of HC **(C)**, FEP and CHR subjects for scale 96 **(D,G)** and 94 **(E,F)** are presented.

The scales and voxels that showed statistically significant differences (corrected with FDR < 0.05) are presented in Figure 7. In Figures 7A,B), the colorbar represents the range of the corrected p-values. For the first comparison, scale 96 across voxels significantly differentiated FEP patients from HC. For the second comparison, scales 94 and 96 across voxels significantly differentiated FEP from CHR patients. This indicates that low frequencies (high scales) are more prominent in FEP, which reflects a smoother divergence of voxels in the spatial-series, i.e. lower structural complexity, compared to both CHR and HC. There was no statistically significant difference in the third comparison. The mean correlation for the specific scales is represented in Figures 7C–G for each group.

**FIGURE 8:**
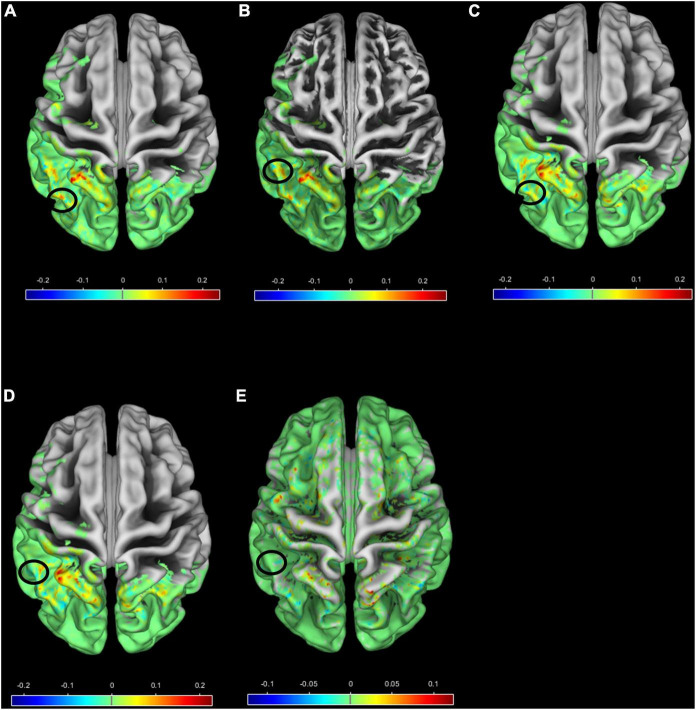
Surface overlay of the mean correlation with Morlet wavelet for **(A)** FEP scale 94, **(B)** FEP scale 96, **(C)** CHR scale 94, **(D)** CHR scale 96 and **(E)** HC scale 96. The color scale represents the range of the correlation, higher (lower) correlation means smooth (sharp) cortical folding, and the black circles indicate regions with different mean correlation between groups **(A)** vs. **(C)**, **(B)** vs. **(D)**, **(B)** vs. **(E)**.

In Figure 8, a surface overlay of the mean correlation of each group with Morlet wavelet is presented for the two scales 94 and 96 using the CAT12 toolbox (for (a) FEP in scale 94, (b) FEP in scale 96, (c) CHR in scale 94, (d) CHR in scale 96, and (e) HC in scale 96). The selected voxels contributing to between-group differences are located in the frontal, occipital, temporal lobe and the cerebellum, involving the rolandic and calcarine areas, the cuneus, the lower parietal lob, the heschl part of cerebellum and vermis. It is observed that not only different regions have different values of correlation but also the same regions (i.e., black circles in Figure 8) folding in a different way compared to the Morlet template in Figures 8A vs. 8C, 8B vs. 8D and 8B vs. 8E. Specifically, for HC subjects in scale 96 the Morlet wavelet is positive correlated (red points in Figure 8) with the lambda values in both hemispheres (Figure 8E). For both scales CHR patients present positive correlation on the right hemisphere (Figures 8C,D) in contrast to FEP that present positive correlation only on the left hemisphere. In Figures 8A-E, the mean correlation of the Morlet wavelet is represented for the voxels with statistical significant differences (corrected p-values showed in Figure 7). It is observed that in scale 96 (scale 94) the correlation with Morlet wavelet is differentiated between FEP and combined CHR and HC (FEP vs. CHR). However, there were no significant differences in gyrification index between all comparisons (FWE < 0.05).

## Discussion

In this study, we investigated gray matter abnormalities in the spatial-scale domain for a large sample of patients with first-episode psychosis (FEP), patients with clinical high risk (CHR) and healthy controls (HC). We applied a combination of established methodologies using lambda and wavelet transformation to identify patients with FEP and CHR. The main advantage of the method is that it requires only 1% of the voxels in GM images; in terms of low-complexity analysis, 5,000 voxels close to the surface or the GM center of the mass were sufficient for the identification of brain topology differences between FEP and CHR against HC, and FEP against CHR.

*First outcome measure:* we considered the nonlinear dynamics of the most weighted voxels by intensity and distance. Two outcome measures were used in this analysis: a) the lambda value and b) the scalograms. Lambda as an outcome measure successfully differentiated FEP and CHR patients from HC but was not sufficient to distinguish FEP from CHR. Multiple findings indicate similar brain abnormalities between CHR and FEP (2, 39–41). Through localization of the top-weighted voxels and multiple comparisons of the lambda across brain regions and groups, statistically significant differences were revealed in the temporal pole, right posterior cingulate gyrus and lingual gyrus and fusiform for FEP against HC, which affected from demographic variables. Statistically significant differences were revealed in the occipital and temporal lobe for CHR against HC, and could serve as biomarkers of psychotic disorders. Many studies present the involvement of the occipital lobe in FEP and CHR (42–44): Subjects with predominant attenuated psychotic symptoms are characterized by a reduction of GM-intensity values in the occipital cortex (44). In previous studies in at-risk individuals progressive gray matter reductions in temporal regions were reported (45–47). The high variability in morphological measures extracted from temporal cortex in schizophrenia was identified in a large meta-analysis (5). Posterior cingulate cortex (45), lingual gyrus (46) and fusiform (47) are reduced in patients with schizophrenia and FEP which is in line with the results presented.

*Second outcome measure*: we considered scalograms of brain sMRI, allowed discrimination of early-stage psychosis from CHR. Both FEP and CHR subjects could be differentiated from HC by simple visual inspection of the scalograms of the lambda extracted from the top 5,000 voxels (see Supplement Figure 4). However, we emphasized on group comparison results. FEP scalograms were statistically significant different from those of HC and CHR; no differences were observed between CHR and HC. Thus, the move from the spatial domain into the frequency domain revealed hidden patterns in the mechanism of the progression of the disease. Two frequencies in the spatial-series of lambda provided the ability to statistically differentiate FEP from CHR, which was not possible using solely the lambda value. The low frequencies presented by high scales was interpreted as smooth changes in the brain topology of FEP compared to CHR and HC. We observed that the nonlinear dynamic of the weighted distances as an expression of the structure relief of the individual brains is highly informative for the identification of brain topology differences in FEP and CHR subjects. These results are consistent with previous studies that showed significant reduction in cortical folding across multiple brain regions, especially left frontal and right temporal regions, in patients with first-episode psychosis compared to healthy controls (48–50). Gyrification index values were significantly increased in the right temporal lobe of the FEP patients compared to HC (51) that is in line with the increased positive correlation in the right temporal lobe for HC subjects in low frequencies, representing smoother cortical folding in HC compared to FEP in this region. Statistically significant differences in the gyrification index are not presented in the SBM analysis for the examined dataset. This fact enhances our notion that chaos analysis provides better insights for the recognition in psychosis and progression of the illness. Abnormal cortical topography was also observed in the occipital lobe in clinical high risk patients in a multi-center study (52), which is consistent with the positive correlation in the occipital lobe for CHR patients. In addition, CHR subjects presented positive correlation in the right occipital lobe in contrast to FEP that presented in the left occipital lobe.

The innovation of the proposed method in the field of psychosis biomarker research is that it uses spatial-series extracted from sMRI, which separates it from other approaches that investigate gray matter volume increase or decrease, such as VBM analysis. Instead, our approach transforms the brain sMRI into a spatial-series, calculates the chaotic gray matter distribution using the lambda value, and finally transforms the lambda series into a two-dimensional (2D) scalogram by using the Wavelet Transform (WT), in order to have a useful representation of spatial-scale features.

The main advantage of the method is that the impact of the initial point of reference, the GM center of mass in individual GM images that was used for the calculation of the distance to voxels, is not reflected in the sorting of the weighted distance and the lambda value. Lambda measures how the distances diverge in the state-space, regarding the distances across all voxels selected, and thus is a ‘path-free’ measurement. As lambda expresses the way that two neighbor voxels, in the state-space, diverge across the GM topology with respect to all the other voxels, it depicts the way that voxels from different regions are related to structural changes in psychosis. As the scales represent the structure relief, it may reflect volume increase patterns in FEP patients compared to CHR subjects. However, our results should be considered in view of certain limitations: The sample size was moderate; moreover, the method contains many parameter selections that warrant further exploration (e.g., the number of the selected voxels). The proposed method uses the higher weighted distance in the brain which are not distinguished between groups and located mostly in the occipital and temporal lobe. This fact enhances our assumption that we do not lose spatial dependences in the spatial signal when sorting, and thus the application of the Largest-Lyapunov-Exponent is feasible. The drawback of the method is the assumption underwent due to computational restrictions and should be find ways to overcome this problem. We plan to address these limitations in further studies investigating the effectiveness and robustness of the method in larger datasets with different scanning parameters, and across different (including non-psychotic) diagnoses. Comparison to classical approaches such as cortical thickness, cortical folding, and gray matter density in addition to the comparison with the gyrification index should be performed in larger datasets to investigate the superiority of the proposed method. It is in author’s future plans to apply explainable AI in a larger database across different diagnoses. Additionally, chaos analysis implementation on multiple modalities of the same sample, i.e., EEGs, and application of the explainable AI on multi-modal prediction models would benefit the identification of potential biomarkers in psychosis. The main outcome of the present study is the identification of brain topology differences of the FEP and CHR patients and it would be of outmost importance to be implemented in the patient management system to support the doctor’s decision.

## Data availability statement

The datasets used and/or analyzed during the current study will be available from the authors upon reasonable request.

## Ethics statement

The studies involving human participants were reviewed and approved by University of Basel. Written informed consent to participate in this study was provided by the participants’ legal guardian/next of kin.

## Author contributions

AK: arise the research question, development of the proposed methodology, interpretation, and writing of the results with the substantial help from all co-authors. MA: support the writing of the results. CA: MRI data acquisition, consultation on the interpretation of the results, and writing of the results. SB: MRI data acquisition, consultation on the interpretation of the results, and writing of the results. TM and HH: consultation on the proposed methodology. All authors contributed to the article and approved the submitted version.

## Footnotes

1
http://dbm.neuro.uni-jena.de
