# Supplementary material for: Chaos analysis of the brain topology in first-episode psychosis and clinical high risk patients
Source: Front Psychiatry. 2022 Oct 13;13:965128. doi: 10.3389/fpsyt.2022.965128 (PMC9606602; doi:10.3389/fpsyt.2022.965128)
Supplement: Supplementary file 1 [file Data_Sheet_1.pdf]

Supplementary Material

Table S1: The mean and std of the center of the mass for each category in x,y,z coordinates.

Coordinates	Mean(std) FEP	Mean(std) CHR	Mean(std) HC
x	60.6710(0.1161)	60.6265 (0.1462)	60.6494 (0.1188)
y	70.1180 (0.5501)	69.9588 (0.5433)	70.1352 (0.6126)
z	53.7673 (0.5483)	53.7328 (0.5500)	53.8205 (0.4254)

Table S2: The mean values of the lambda in brain regions were calculated using the aal.nii template in the MRIcron.

Regions	HC	FEP	CHR	Regions	HC	FEP	CHR
**Precentral_L 2001**	0.003022	0.000279	0.000676	**Insula_R 3002**	0.004949	-	-
**Precentral_R 2002**	0.00295	0.00011	-	**Cingulum_Ant_L 4001**	0.005741	-	-
**Frontal_Sup_L 2101**	0.004775	0.000182	6.5E-05	**Cingulum_Ant_R 4002**	0.006448	-	-
**Frontal_Sup_R 2102**	0.00439	0.000146	-	**Cingulum_Mid_L 4011**	0.006579	0.000381	0.0009
**Frontal_Sup_Orb_L 2111**	0.003596	3.25E-05	-	**Cingulum_Mid_R 4012**	0.006386	0.000217	0.000671
**Frontal_Sup_Orb_R 2112**	0.003407	-	-	**Cingulum_Post_L 4021**	0.01233	0.00025	0.00204
**Frontal_Mid_L 2201**	0.006401	0.000603	0.000248	**Cingulum_Post_R 4022**	0.013168	0.000274	0.00096
**Frontal_Mid_R 2202**	0.005778	-	-	**Hippocampus_L 4101**	0.012776	0.00018	0.000203
**Frontal_Mid_Orb_L 2211**	0.002648	0.000143	3.13E-05	**Hippocampus_R 4102**	0.011463	2.48E-05	3.32E-05
**Frontal_Mid_Orb_R 2212**	0.001348	-	-	**ParaHippocampal_L 4111**	0.010331	0.000457	0.001008
**Frontal_Inf_Oper_L 2301**	0.004043	0.001904	0.001281	**ParaHippocampal_R 4112**	0.009948	4.1E-05	0.000161
**Frontal_Inf_Oper_R 2302**	0.004451	-	-	**Amygdala_L 4201**	0.0076	5.39E-05	0.000139
**Frontal_Inf_Tri_L 2311**	0.004243	0.001132	0.000718	**Amygdala_R 4202**	0.005232	-	-
**Frontal_Inf_Tri_R 2312**	0.004118	-	-	**Calcarine_L 5001**	0.016066	0.012072	0.020314
**Frontal_Inf_Orb_L 2321**	0.003289	0.000187	0.000209	**Calcarine_R 5002**	0.01548	0.007183	0.011624
**Frontal_Inf_Orb_R 2322**	0.003643	-	-	**Cuneus_L 5011**	0.008546	0.010787	0.014409
**Rolandic_Oper_L 2331**	0.00495	0.001121	0.002908	**Cuneus_R 5012**	0.008534	0.007826	0.009961
**Rolandic_Oper_R 2332**	0.003859	-	-	**Lingual_L 5021**	0.022632	0.014612	0.023411
**Supp_Motor_Area_L 2401**	0.002753	4.42E-05	6.41E-05	**Lingual_R 5022**	0.021299	0.009883	0.012685
**Supp_Motor_Area_R 2402**	0.002055	2.48E-05	-	**Occipital_Sup_L 5101**	0.00756	0.01796	0.024234

**Olfactory_L 2501**	0.005898	2.48E-05	2.8E-05	**Occipital_Sup_R 5102**	0.008324	0.007679	0.009451
**Olfactory_R 2502**	0.005827	-	-	**Occipital_Mid_L 5201**	0.011682	0.050776	0.061928
**Frontal_Sup_Medial_L 2601**	0.006665	-	-	**Occipital_Mid_R 5202**	0.009748	0.012182	0.013599
**Frontal_Sup_Medial_R 2602**	0.005431	-	-	**Occipital_Inf_L 5301**	0.024699	0.070364	0.08431
**Frontal_Med_Orb_L 2611**	0.003935	-	-	**Occipital_Inf_R 5302**	0.021499	0.025263	0.032682
**Frontal_Med_Orb_R 2612**	0.003828	-	-	**Fusiform_L 5401**	0.01742	0.023794	0.029127
**Rectus_L 2701**	0.003427	-	-	**Fusiform_R 5402**	0.016982	0.005035	0.00749
**Rectus_R 2702**	0.003016	-	-	**Postcentral_L 6001**	0.003181	0.001857	0.002642
**Insula_L 3001**	0.005417	0.000246	0.000261	**Postcentral_R 6002**	0.002635	0.000217	7.78E-05
**Pallidum_L 7021**	0.006273	-	-	**Parietal_Sup_L 6101**	0.002784	0.011137	0.012026
**Pallidum_R 7022**	0.006175	-	-	**Parietal_Sup_R 6102**	0.001669	0.002149	0.002238
**Thalamus_L 7101**	0.006496	4.57E-05	-	**Parietal_Inf_L 6201**	0.005354	0.018019	0.018865
**Thalamus_R 7102**	0.006587	6E-05	-	**Parietal_Inf_R 6202**	0.002996	0.000367	0.00086
**Heschl_L 8101**	0.007117	0.0012	0.002301	**SupraMarginal_L 6211**	0.01082	0.018919	0.025537
**Heschl_R 8102**	0.006208	-	-	**SupraMarginal_R 6212**	0.009314	0.000334	0.000358
**Temporal_Sup_L 8111**	0.009322	0.010986	0.014439	**Angular_L 6221**	0.006415	0.050732	0.045735
**Temporal_Sup_R 8112**	0.009387	0.000422	0.00048	**Angular_R 6222**	0.00791	0.002605	0.003489
**Temporal_Pole_Sup_L 8121**	0.005783	0.00033	0.001119	**Precuneus_L 6301**	0.005223	0.00427	0.006183
**Temporal_Pole_Sup_R 8122**	0.005061	-	-	**Precuneus_R 6302**	0.006328	0.002465	0.003439
**Temporal_Mid_L 8201**	0.01431	0.041867	0.04809	**Paracentral_Lobule_L 6401**	0.001094	0.000107	8.43E-05
**Temporal_Mid_R 8202**	0.015075	0.004775	0.005413	**Paracentral_Lobule_R 6402**	0.001556	9.26E-05	7.75E-05
**Temporal_Pole_Mid_L 8211**	0.008894	0.00081	0.003322	**Caudate_L 7001**	0.004961	-	-
**Temporal_Pole_Mid_R 8212**	0.008013	5.03E-05	-	**Caudate_R 7002**	0.004719	-	-
**Temporal_Inf_L 8301**	0.012557	0.03438	0.039589	**Putamen_L 7011**	0.005168	-	-
**Temporal_Inf_R 8302**	0.014634	0.005661	0.006877	**Putamen_R 7012**	0.004762	-	-
**Cerebelum_Crus1_L 9001**	0.016552	0.032345	0.032591	**Cerebelum_8_R 9062**	0.02861	0.010529	0.00875
**Cerebelum_Crus1_R 9002**	0.015734	0.006975	0.007177	**Cerebelum_9_L 9071**	0.020917	0.00804	0.006176

**Cerebelum_Crus2_L 9011**	0.023158	0.043888	0.030374	**Cerebelum_9_R 9072**	0.023733	0.006007	0.004483
**Cerebelum_Crus2_R 9012**	0.021639	0.014478	0.010157	**Cerebelum_10_L 9081**	0.003962	0.000733	3.71E-05
**Cerebelum_3_L 9021**	0.012427	-2.6E-06	0.000145	**Cerebelum_10_R 9082**	0.005034	5.6E-05	4.65E-05
**Cerebelum_3_R 9022**	0.01509	4.46E-06	-0.00014	**Vermis_1_2 9100**	0.015472	3.16E-05	-
**Cerebelum_4_5_L 9031**	0.015181	0.000962	0.001436	**Vermis_3 9110**	0.018896	-2.2E-05	-0.00023
**Cerebelum_4_5_R 9032**	0.017649	0.000109	-0.0005	**Vermis_4_5 9120**	0.021933	0.000439	-0.00032
**Cerebelum_6_L 9041**	0.018086	0.018369	0.016322	**Vermis_6 9130**	0.027208	0.002632	0.000374
**Cerebelum_6_R 9042**	0.017176	0.002375	0.002879	**Vermis_7 9140**	0.023338	0.004299	-0.00022
**Cerebelum_7b_L 9051**	0.026212	0.037248	0.027563	**Vermis_8 9150**	0.023322	0.006177	0.000459
**Cerebelum_7b_R 9052**	0.026884	0.013115	0.014945	**Vermis_9 9160**	0.013816	0.002371	0.00179
**Cerebelum_8_L 9061**	0.027044	0.021846	0.018316	**Vermis_10 9170**	0.009819	0.000489	0.000472

Table S3: MNI coordinates of the clusters for the comparison FEP>HC.

Location	x/y/z (mm)	P_{FWE_corr}	P_{FDR_corr}	T_score	Cluster size
Temporal pole	-54/-42/-12	0.000	0.000	8.09	4
FuG	-42/-54/-9	0.002	0.146 (0.004 in cluster level)	6.02	6
LiG	-21.7/-67.5/-13.12	0.000	0.000	5.69	4
Right PCgG	4.7/-40.0/5	0.005	0.112 (0.008 in cluster level)	6.12	5

Table S4: MNI coordinates of the clusters for the comparison CHR>HC.

Location	x/y/z (mm)	P_{FWE_corr}	P_{FDR_corr}	T_score	Cluster size
Temporal pole	-54/-68/6	<0.000	<0.046	6.88	13
Occipital pole	-51/-62/5	<0.0100	<0.046	6.83	8

Table S5: Statistical results of the VBM analysis for the contrast FEP>HC.

Location	x/y/z (mm)	P_{FWE_corr}	P_{FDR_corr}	T_score	Cluster size
Right Cerebellum	39/-62/-35	<0.000	<0.000	9.47	917

Left Cerebellum Exterior	-39/-63/-33	<0.000	<0.001	7.03	131

Figure S1: The histogram of the voxel's intensity for all the warped and modulated images from all subjects and a zoom representation for the high intensities.

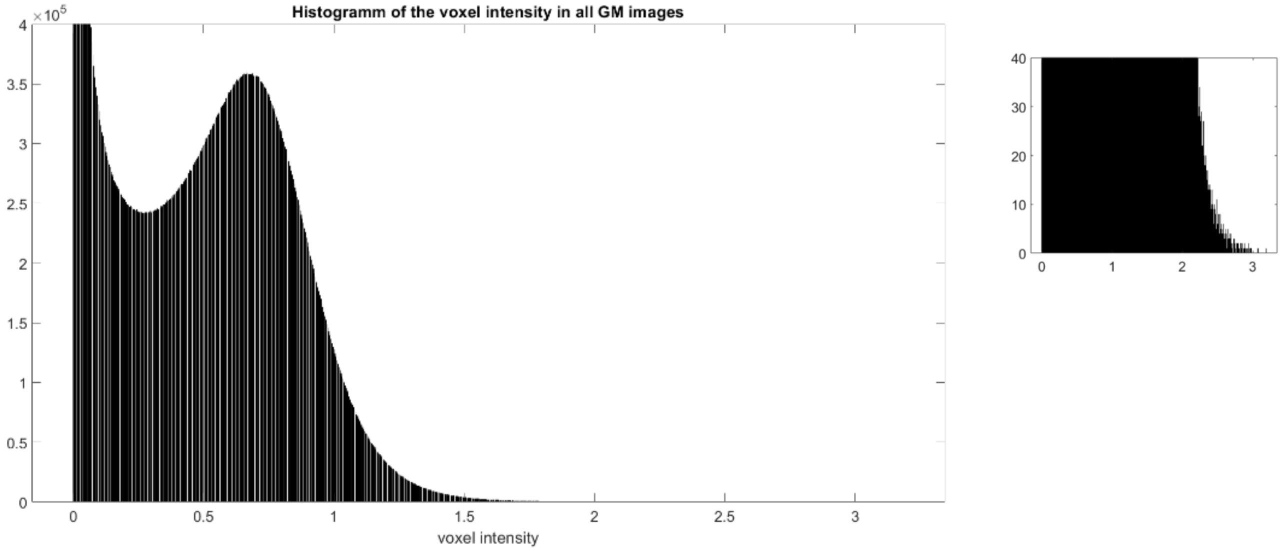

Figure S2: The distances from one HC subject are represented in a) and the weighted distances of the same subject in b). Both mapped in mwp1* image of the same subject. It is observed that the highest distances are located in the temporal and occipital lobe (red color). In c) the upper graph (117.119) shows the distance at the middle frontal gyrus and the lower graph shows the weighted distance (160.431). In d) the left cerebellum exterior presents distance equal to 193.315 (upper graph) and weighted distance equal to 122.072 (lower graph).

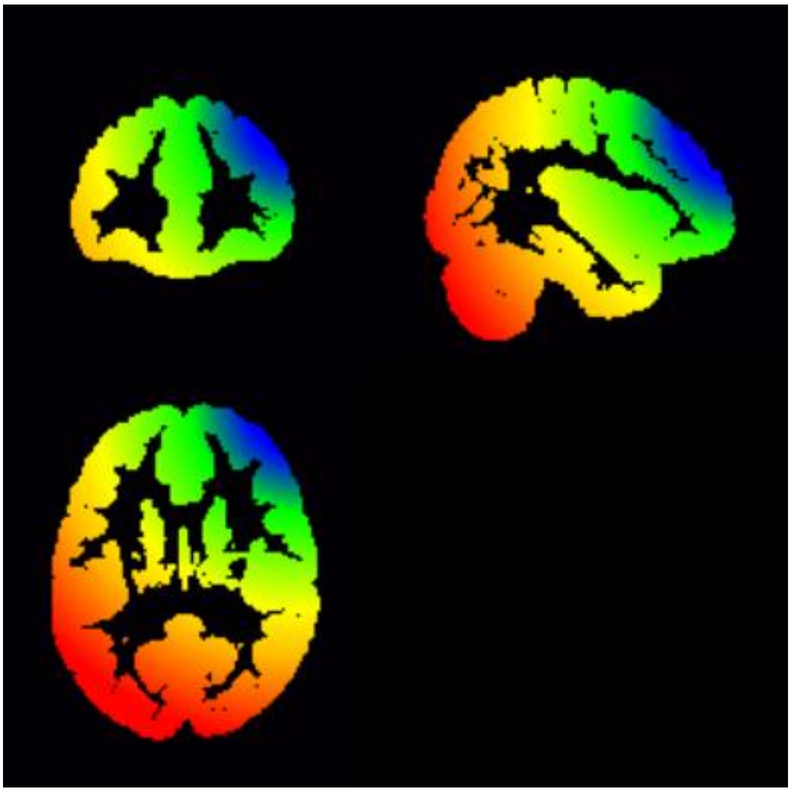

a)

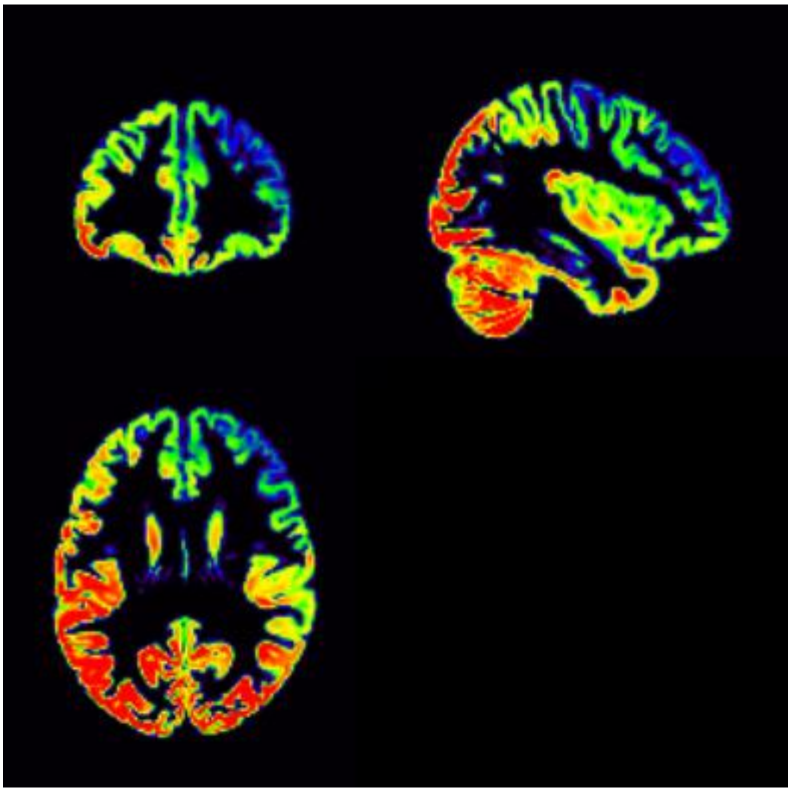

b)

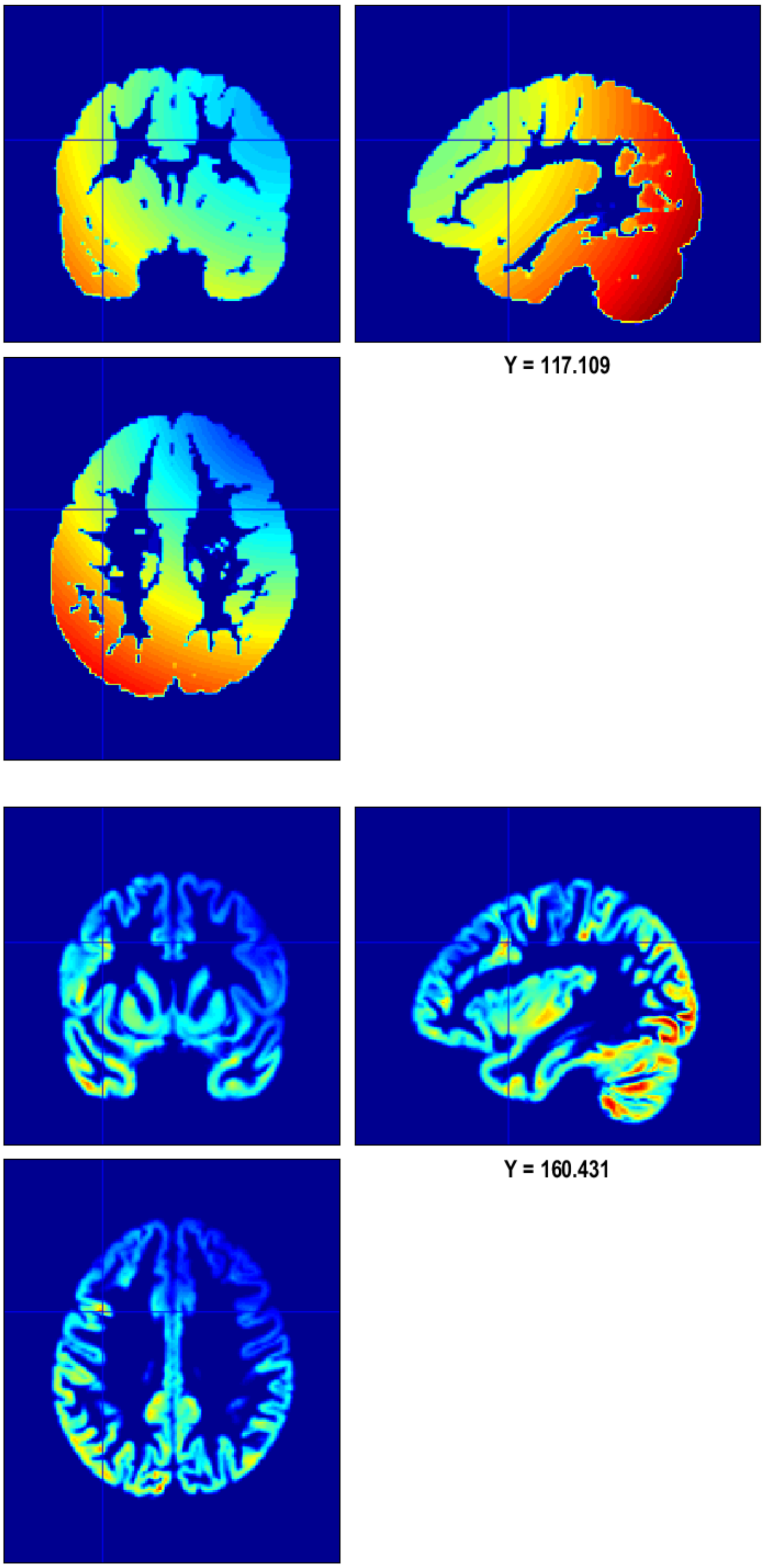

c)

d)

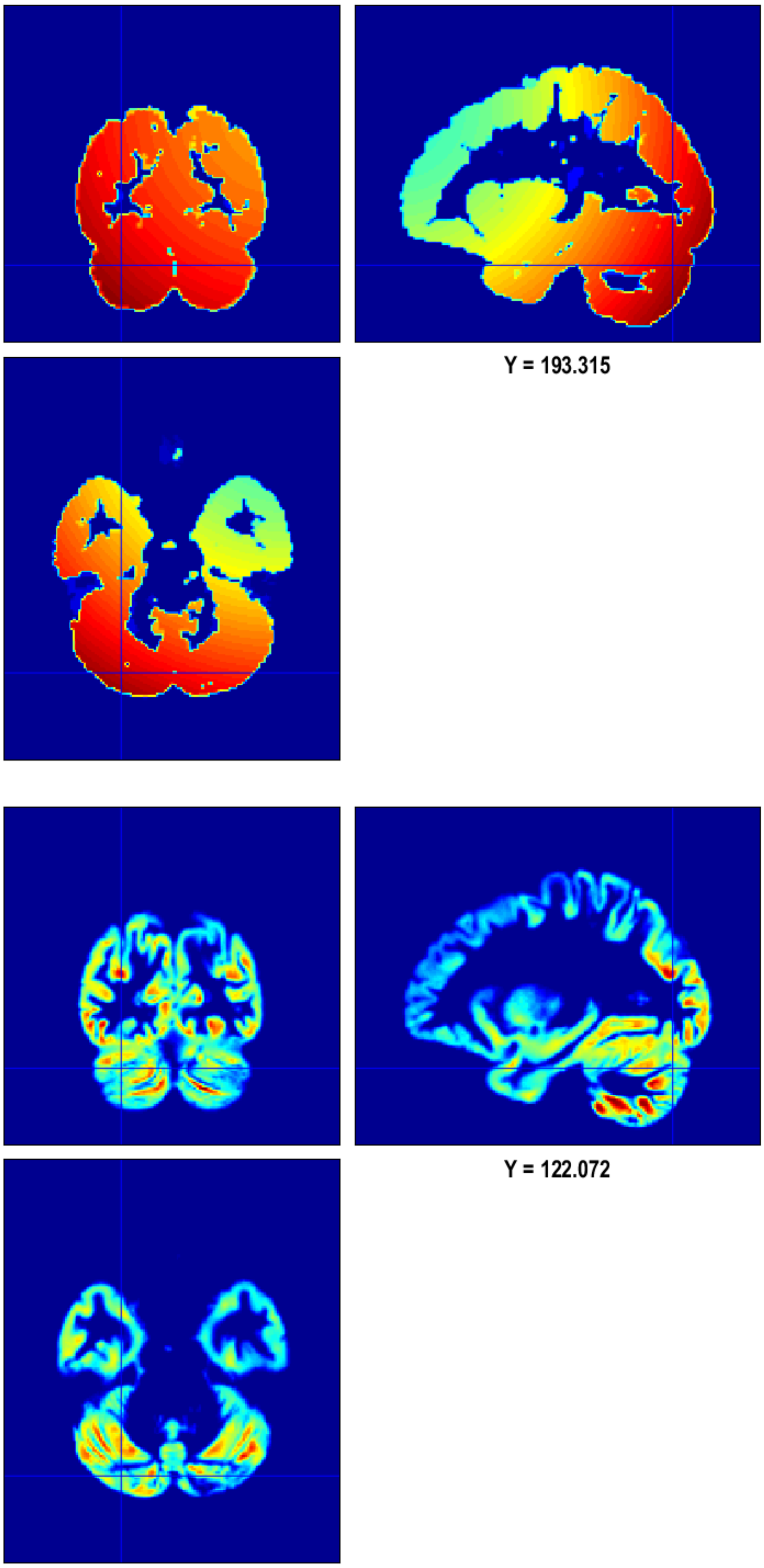

d)

Figure S3: Scalogram of one a) FEP, b) CHR and c) HC subject for 200 scales and 100 scales. Sampled voxels are represented in x-axis and scales in y-axis, the colors represent the correlation with the Morlet wavelet deep red color corresponds to lower and white color to higher correlation.

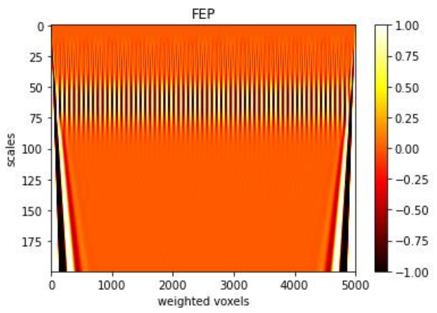

a)

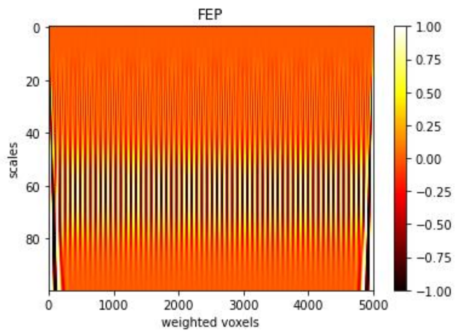

b)

sa

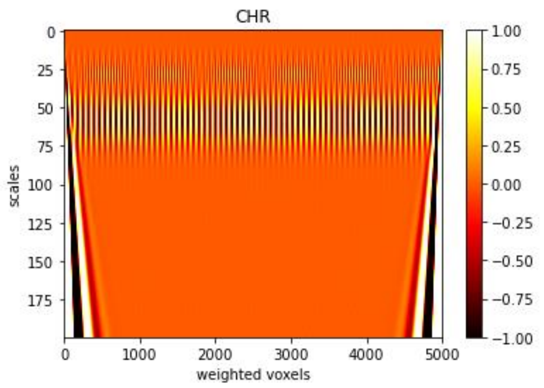

c)s

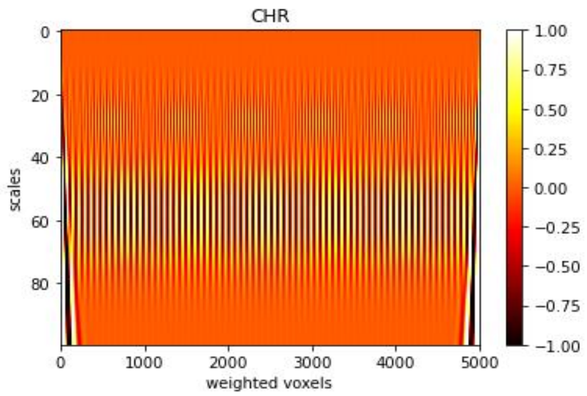

d)

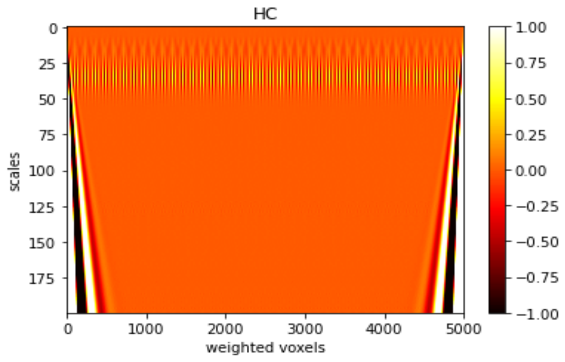

e)

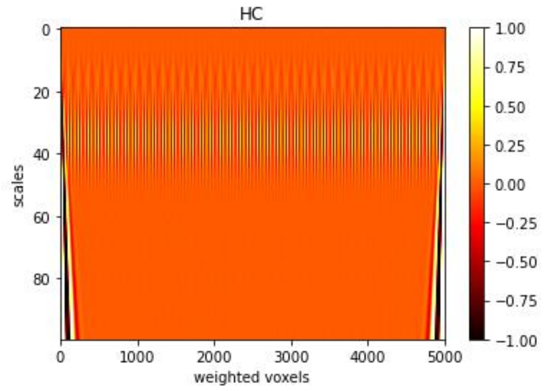

f)

sampled voxels

Figure S4: Individual scalograms of all analyzed FEP patients, CHR and HC subjects, deep red color corresponds to lower and white color to higher correlation.

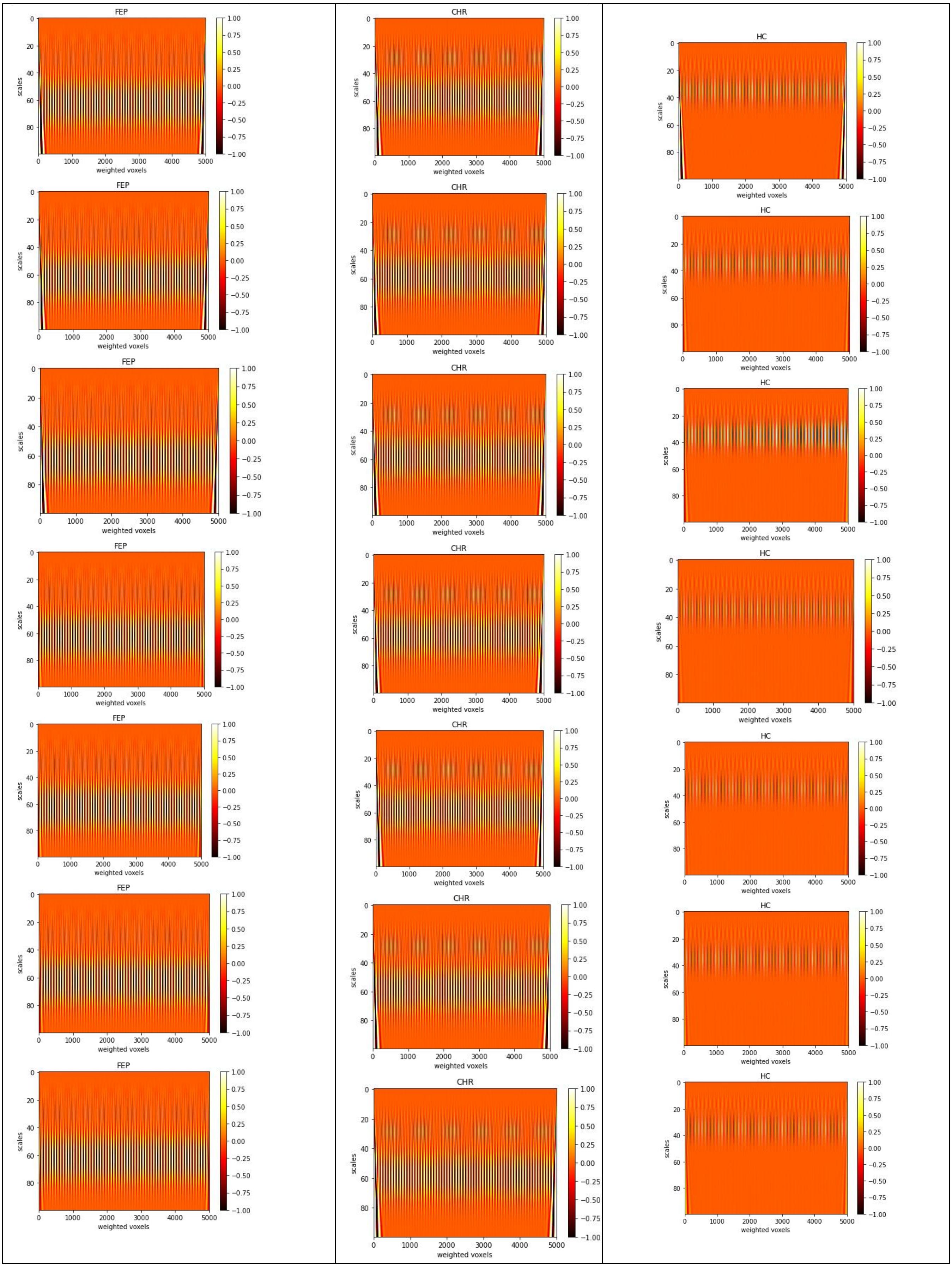
FEP
CHR
HC
scales
weighted voxels
0
20
40
60
80
1000
2000
3000
4000
5000
1.00
0.75
0.50
0.25
0.00
−0.25
−0.50
−0.75
−1.00

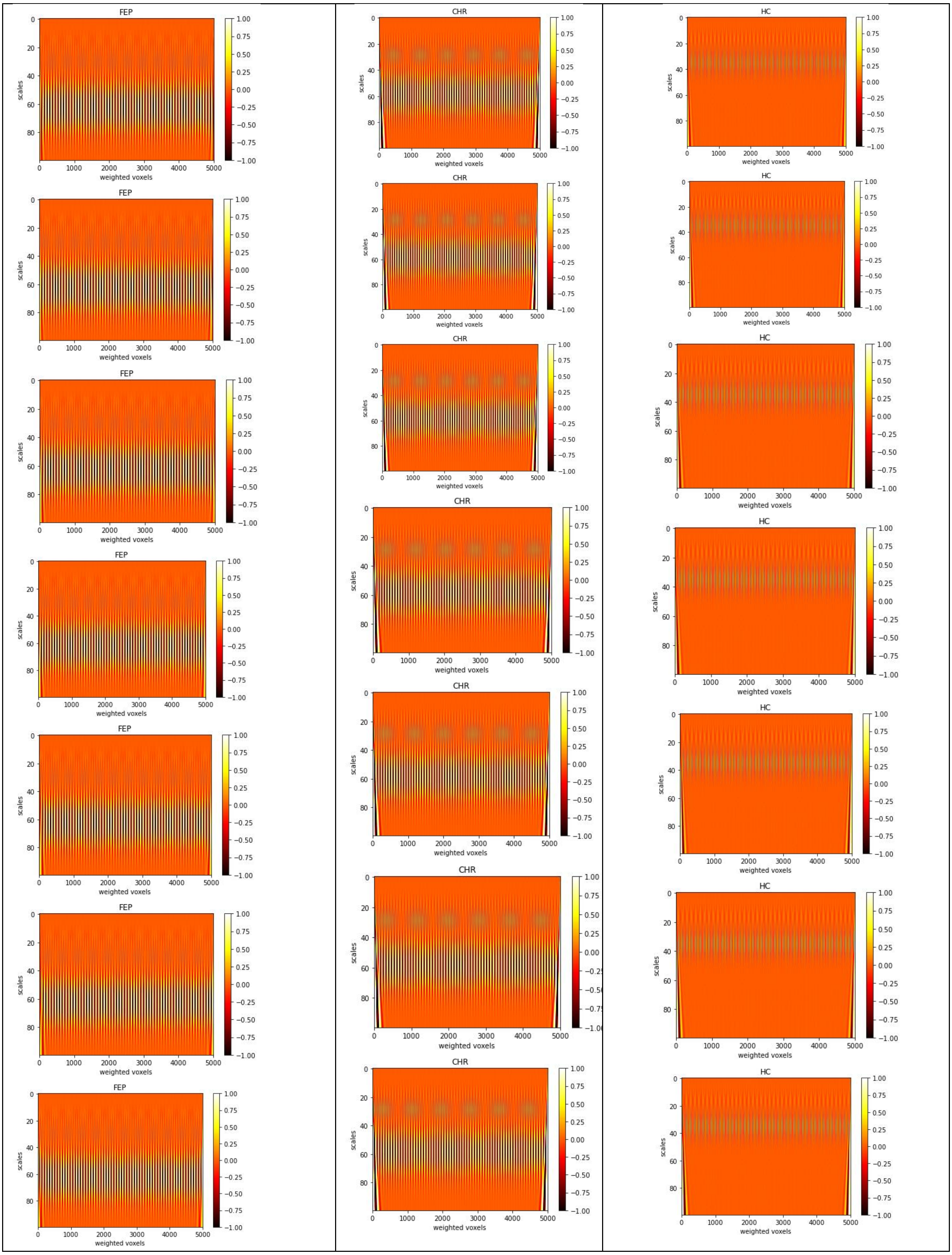
FEP
CHR
HC
scales
weighted voxels
0
20
40
60
80
0
1000
2000
3000
4000
5000
1.00
0.75
0.50
0.25
0.00
−0.25
−0.50
−0.75
−1.00

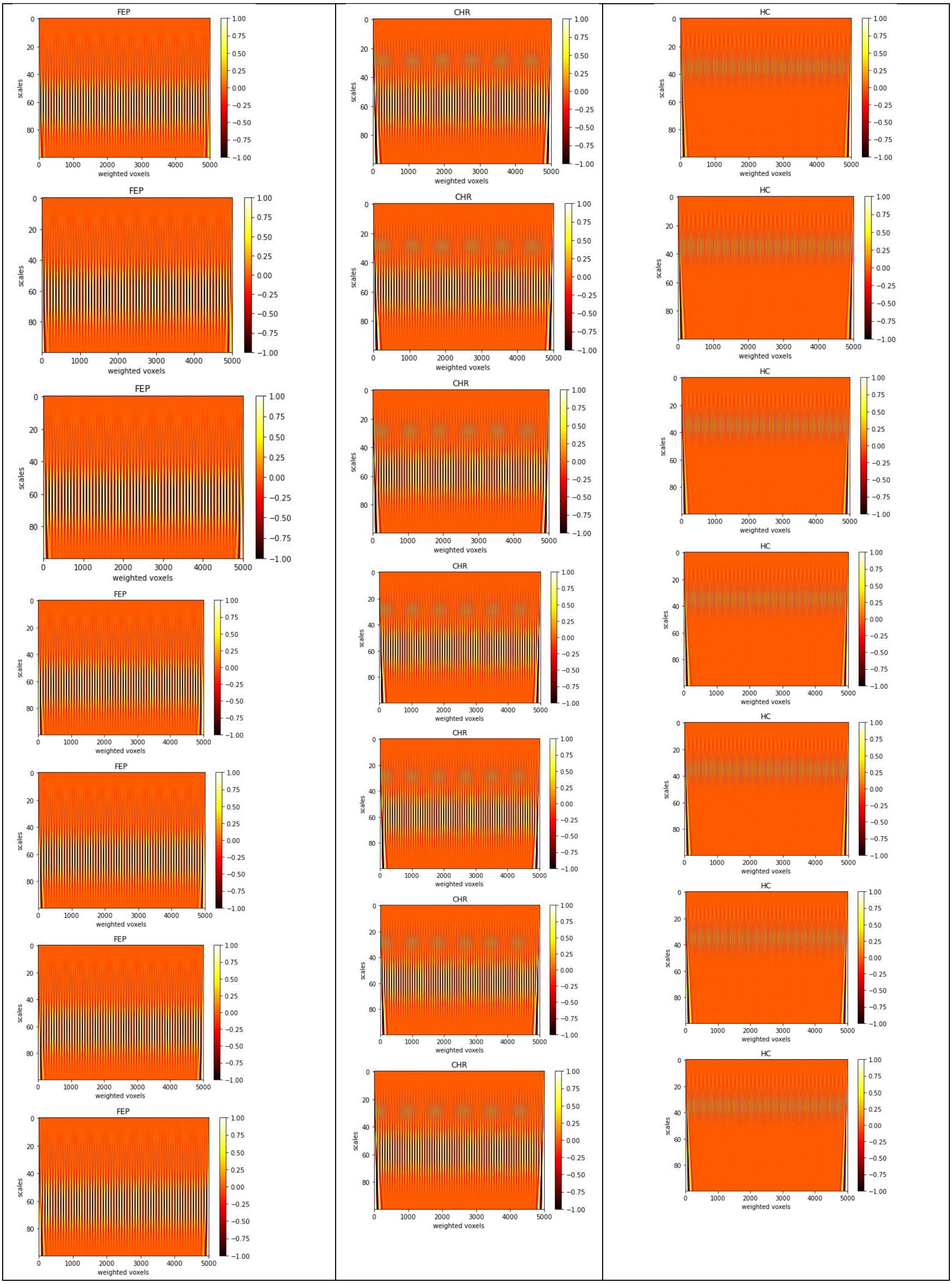
FEP
CHR
HC
scales
weighted voxels
0 20 40 60 80
0 1000 2000 3000 4000 5000
1.00 0.75 0.50 0.25 0.00 −0.25 −0.50 −0.75 −1.00

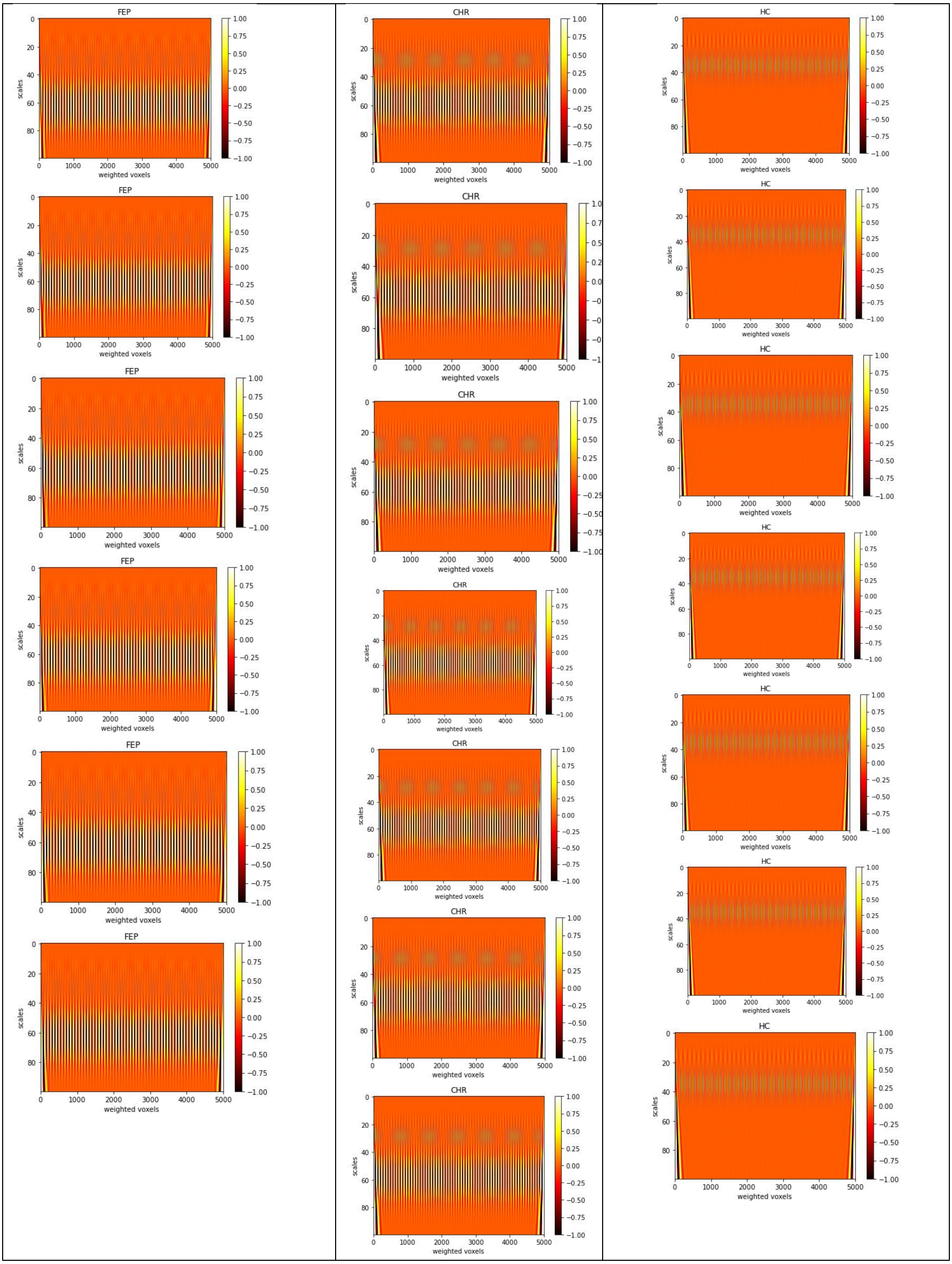
FEP
CHR
HC
scales
weighted voxels
0
20
40
60
80
1000
2000
3000
4000
5000
1.00
0.75
0.50
0.25
0.00
−0.25
−0.50
−0.75
−1.00

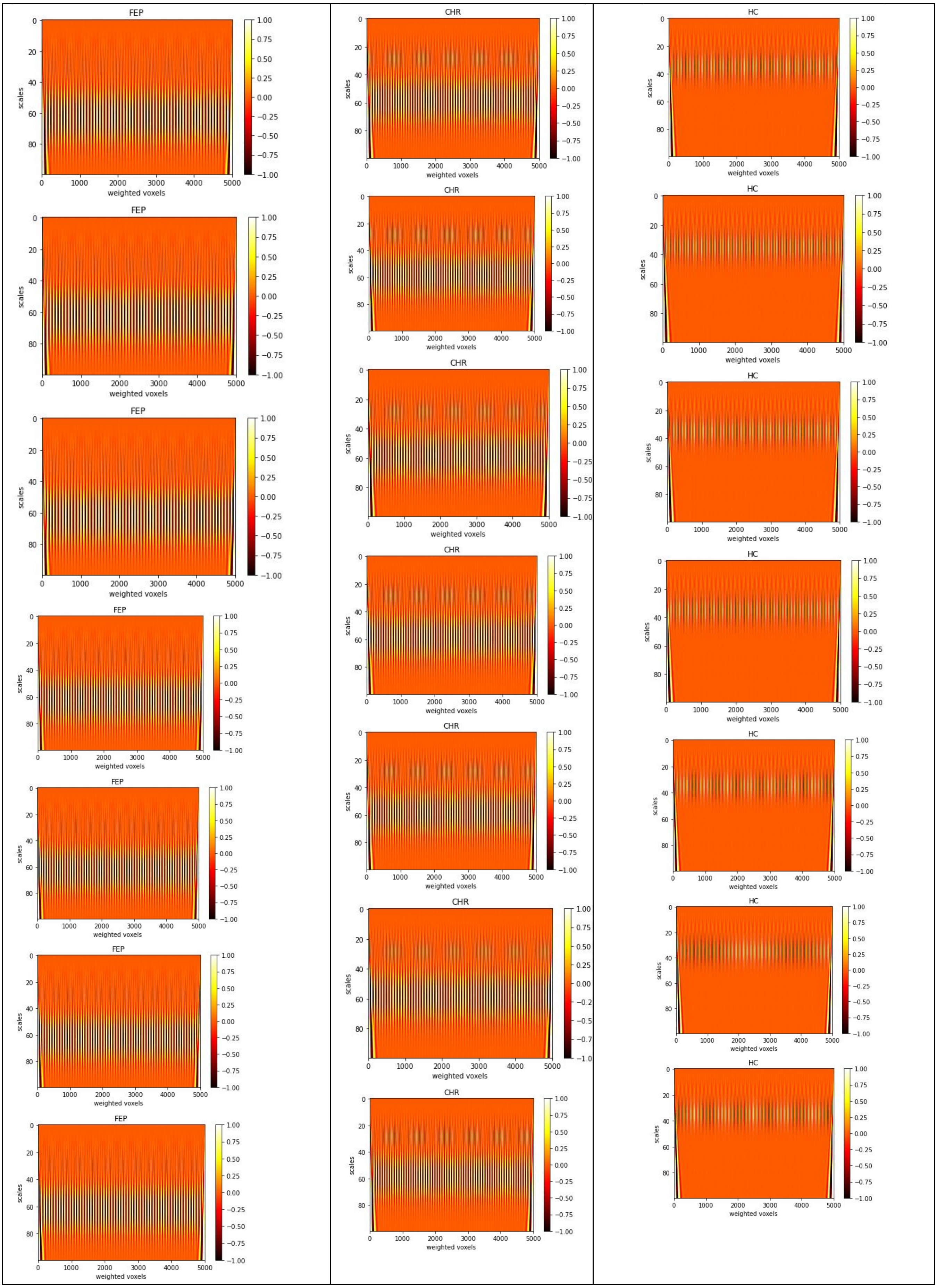
FEP
CHR
HC
scales
weighted voxels

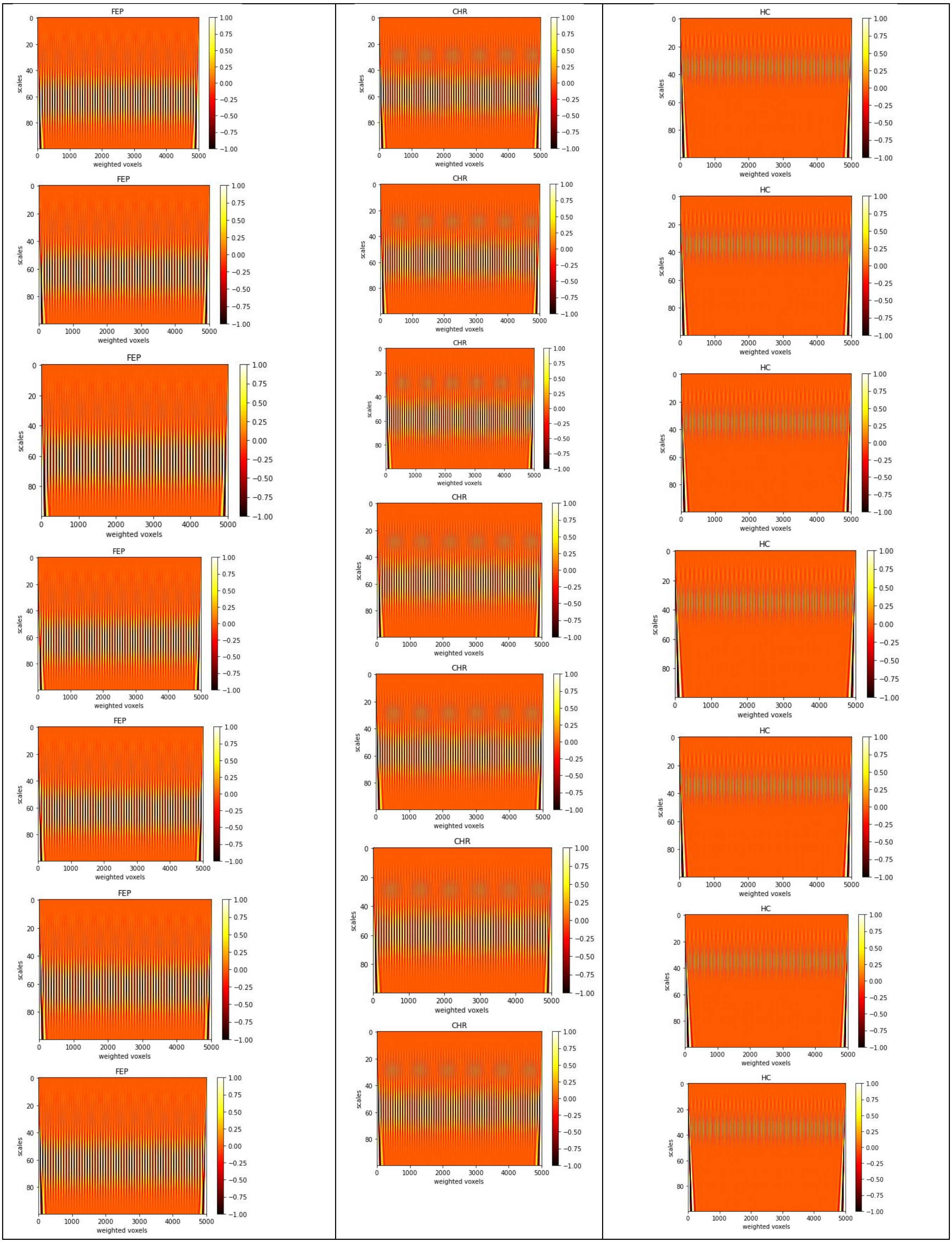
FEP
CHR
HC
scales
weighted voxels

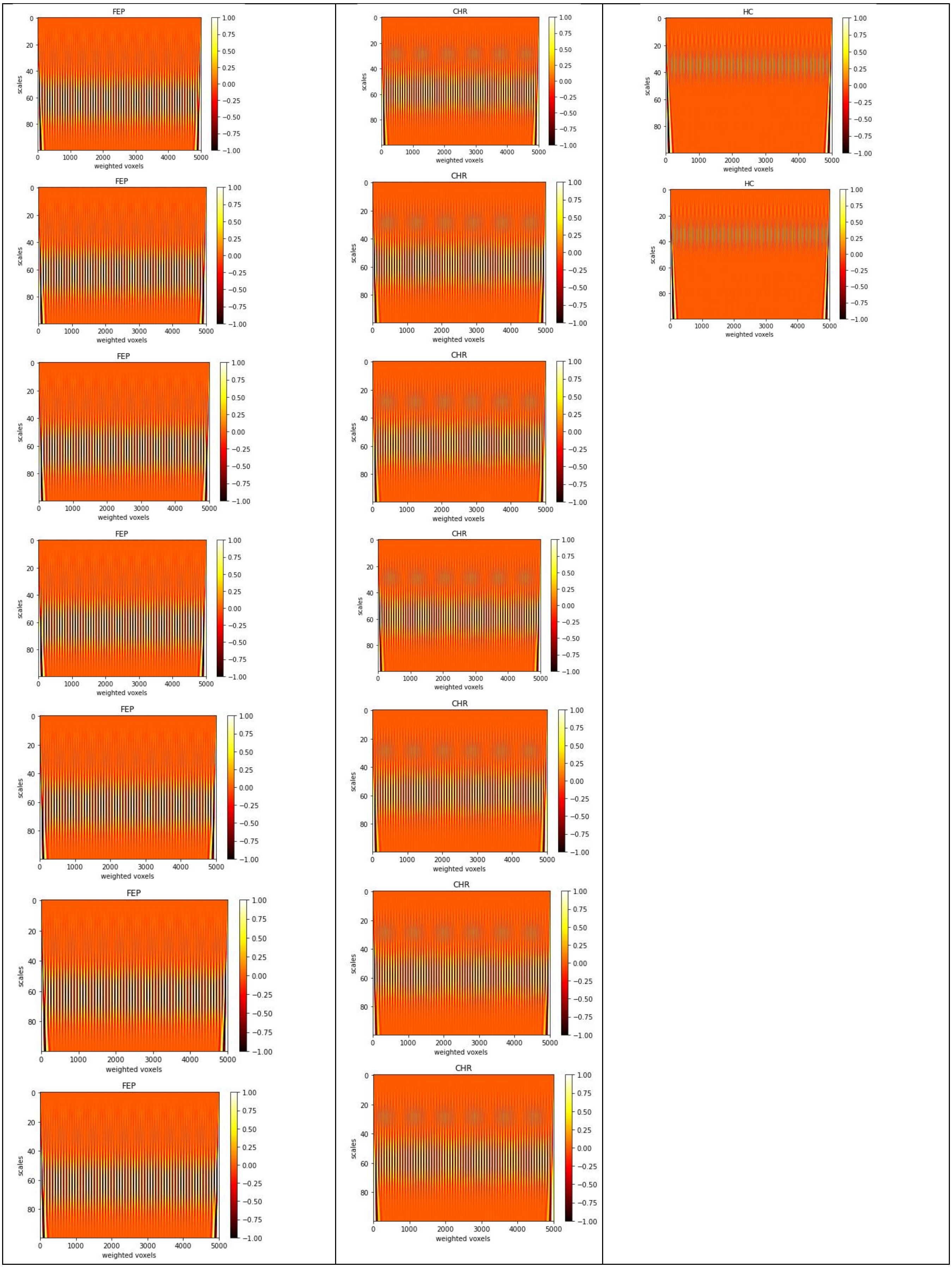
FEP
CHR
HC
scales
weighted voxels
0
20
40
60
80
1000
2000
3000
4000
5000
1.00
0.75
0.50
0.25
0.00
−0.25
−0.50
−0.75
−1.00

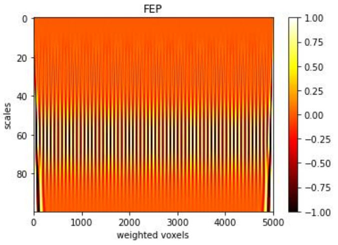
FEP
scales
weighted voxels

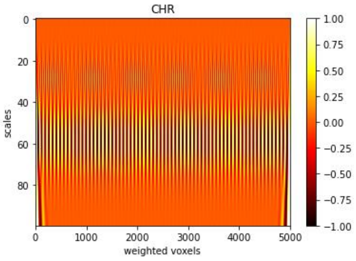
CHR
scales
weighted voxels

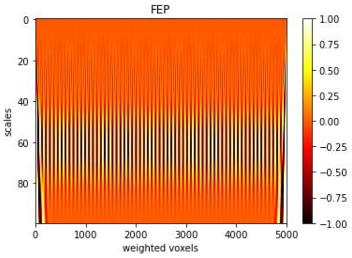
FEP
scales
weighted voxels

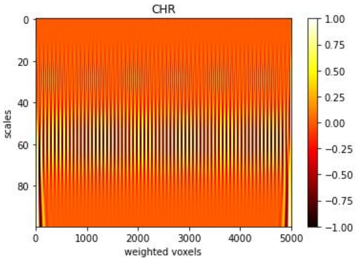
CHR
scales
weighted voxels

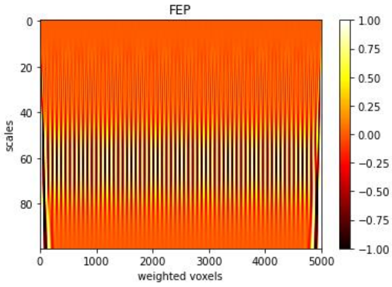
FEP
scales
weighted voxels

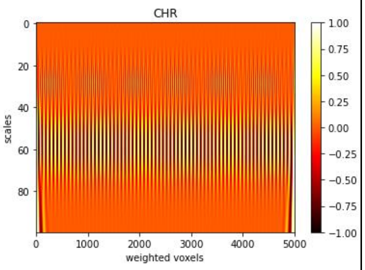
CHR
scales
weighted voxels

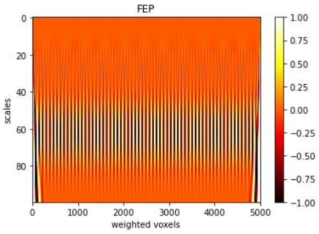
FEP
scales
weighted voxels

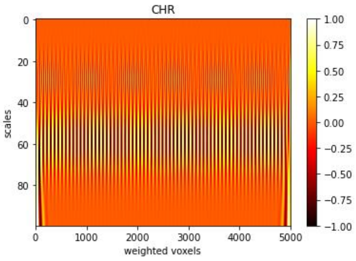
CHR
scales
weighted voxels

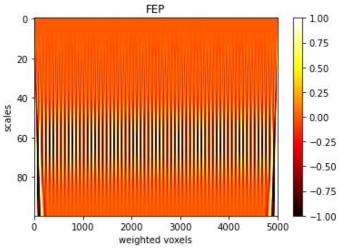
FEP
scales
weighted voxels

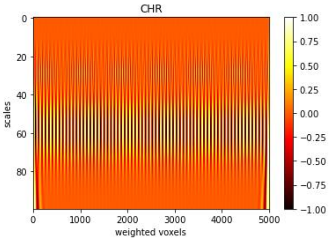
CHR
scales
weighted voxels

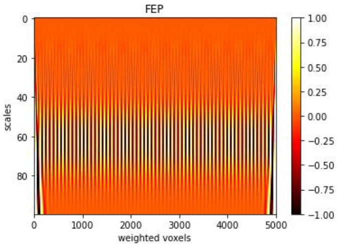
FEP
scales
weighted voxels

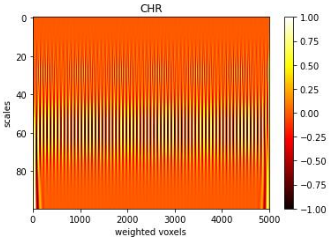
CHR
scales
weighted voxels

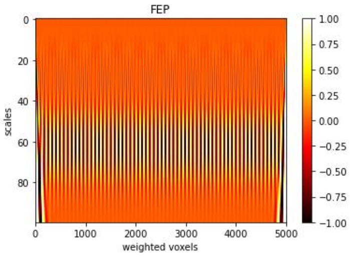
FEP
scales
weighted voxels

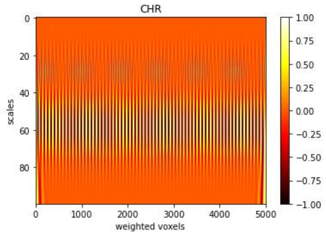
CHR
scales
weighted voxels

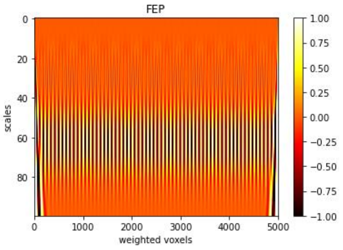
FEP
scales
weighted voxels
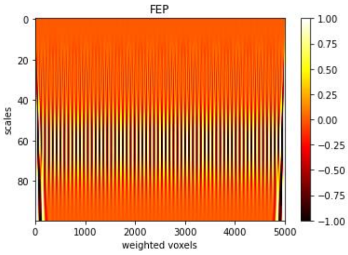
FEP
scales
weighted voxels
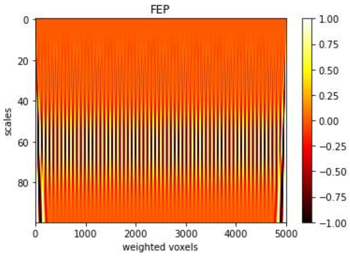
FEP
scales
weighted voxels
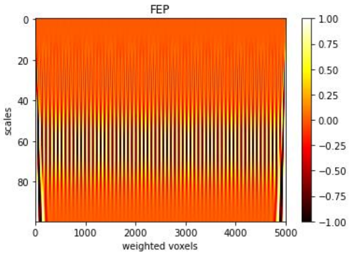
FEP
scales
weighted voxels
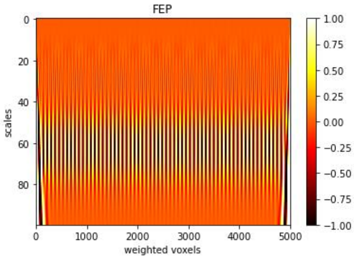
FEP
scales
weighted voxels
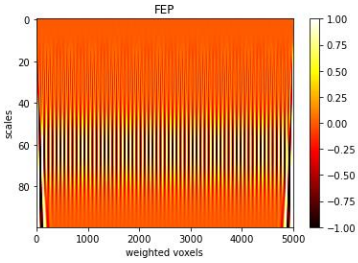
FEP
scales
weighted voxels
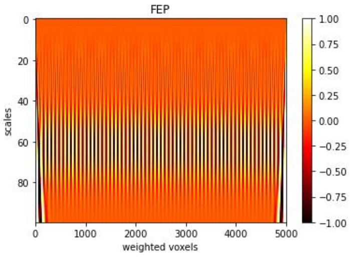
FEP
scales
weighted voxels
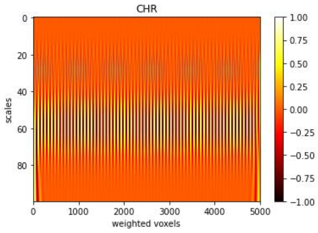
CHR
scales
weighted voxels
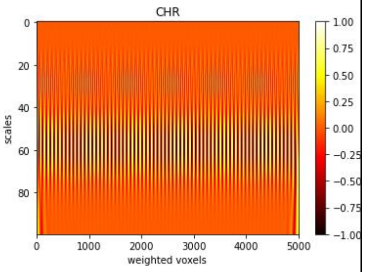
CHR
scales
weighted voxels
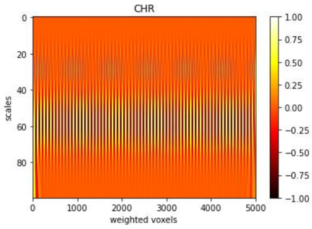
CHR
scales
weighted voxels
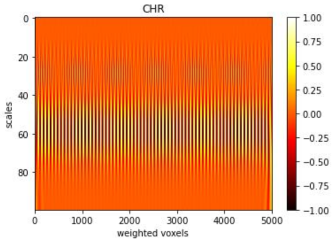
CHR
scales
weighted voxels
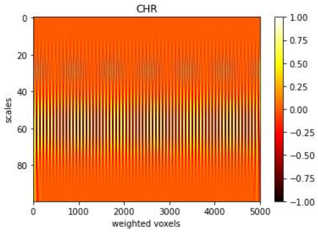
CHR
scales
weighted voxels
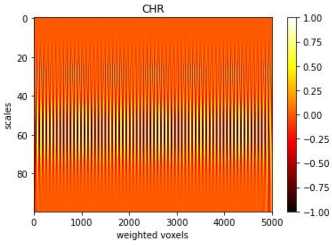
CHR
scales
weighted voxels
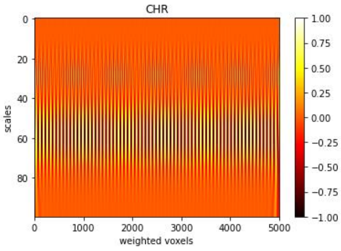
CHR
scales
weighted voxels

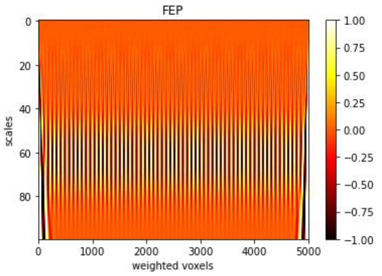
FEP
scales
weighted voxels

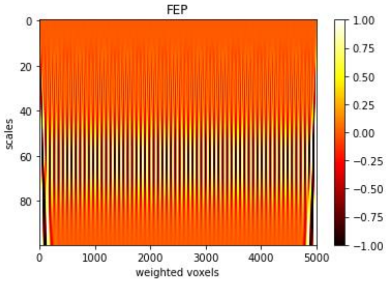
FEP
scales
weighted voxels

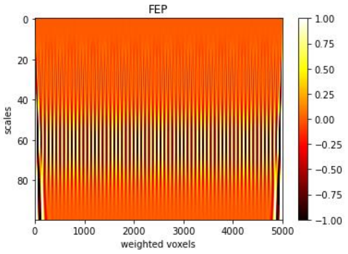
FEP
scales
weighted voxels

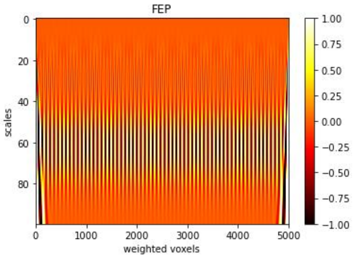
FEP
scales
weighted voxels

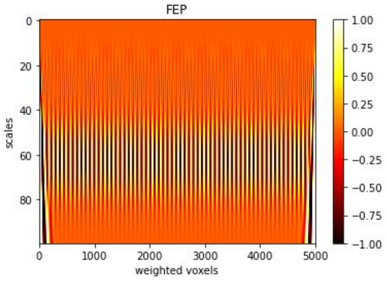
FEP
scales
weighted voxels

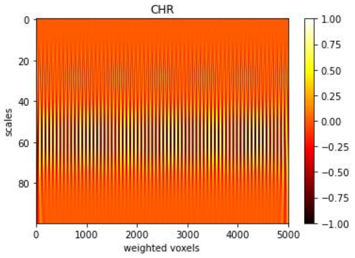
CHR
scales
weighted voxels

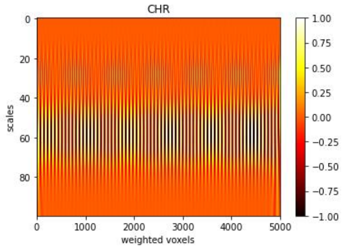
CHR
scales
weighted voxels

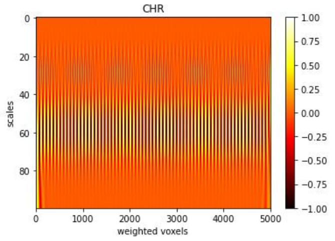
CHR
scales
weighted voxels

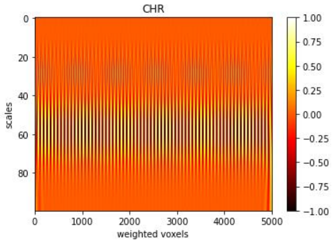
CHR
scales
weighted voxels

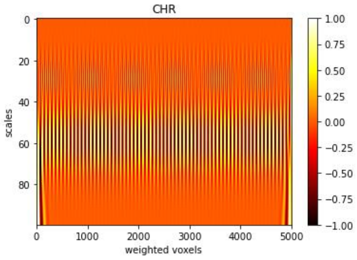
CHR
scales
weighted voxels

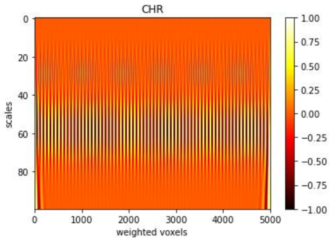
CHR
scales
weighted voxels

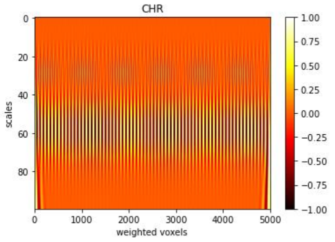
CHR
scales
weighted voxels

<table>
<tr>
<td>
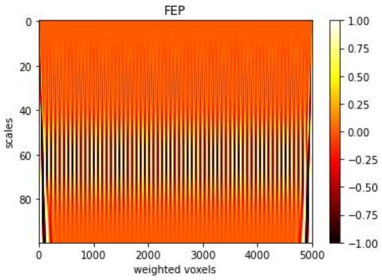

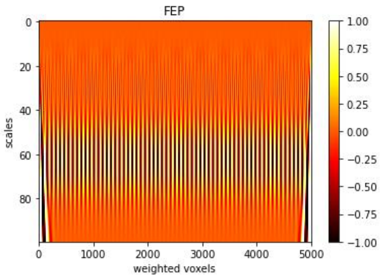

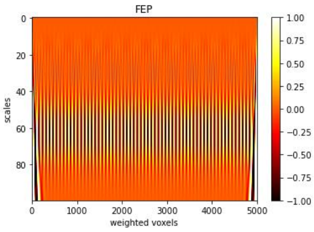

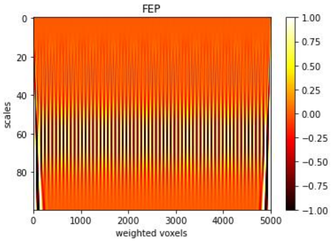

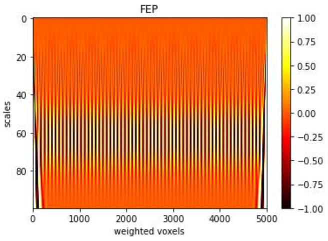

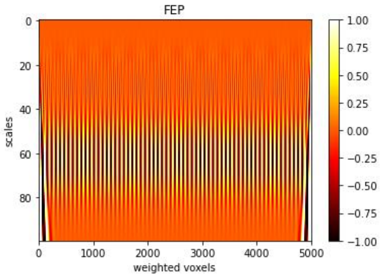

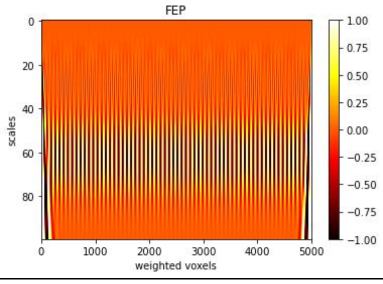

</td>
<td>
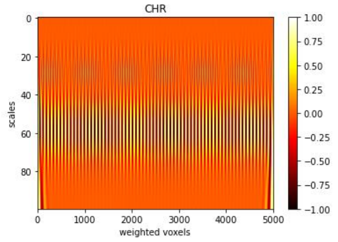

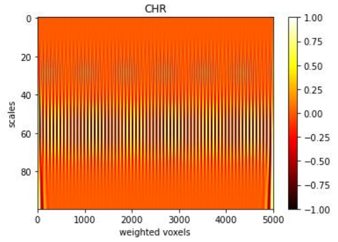

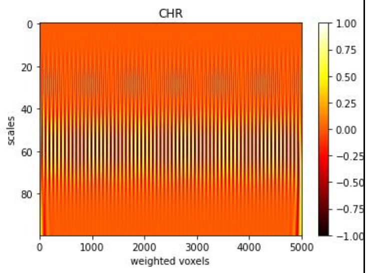

</td>
<td></td>
</tr>
</table>

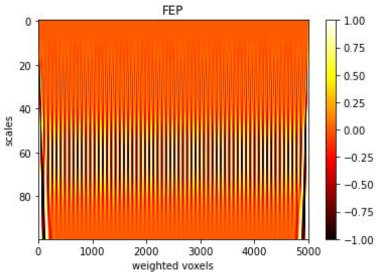FEP<br>scales<br>weighted voxels<br>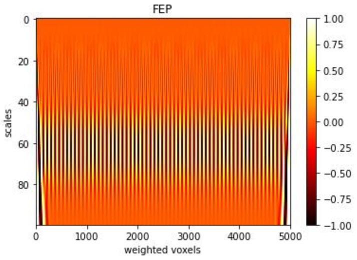FEP<br>scales<br>weighted voxels<br>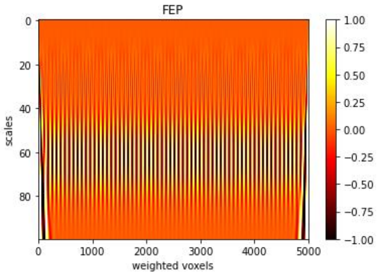FEP<br>scales<br>weighted voxels		
